# Neurocomputational mechanisms underlying subjective valuation of effort costs

**DOI:** 10.1371/journal.pbio.1002598

**Published:** 2017-02-24

**Authors:** Trevor T.-J. Chong, Matthew Apps, Kathrin Giehl, Annie Sillence, Laura L. Grima, Masud Husain

**Affiliations:** 1 Monash Institute of Cognitive and Clinical Neurosciences, Monash University, Melbourne, Australia; 2 Department of Experimental Psychology, University of Oxford, Oxford, United Kingdom; 3 Nuffield Department of Clinical Neurosciences, John Radcliffe Hospital, Oxford, United Kingdom; 4 Department of Nuclear Medicine, University of Cologne, Cologne, Germany; University of Cambridge, UNITED KINGDOM

## Abstract

In everyday life, we have to decide whether it is worth exerting effort to obtain rewards. Effort can be experienced in different domains, with some tasks requiring significant cognitive demand and others being more physically effortful. The motivation to exert effort for reward is highly subjective and varies considerably across the different domains of behaviour. However, very little is known about the computational or neural basis of how different effort costs are subjectively weighed against rewards. Is there a common, domain-general system of brain areas that evaluates all costs and benefits? Here, we used computational modelling and functional magnetic resonance imaging (fMRI) to examine the mechanisms underlying value processing in both the cognitive and physical domains. Participants were trained on two novel tasks that parametrically varied either cognitive or physical effort. During fMRI, participants indicated their preferences between a fixed low-effort/low-reward option and a variable higher-effort/higher-reward offer for each effort domain. Critically, reward devaluation by both cognitive and physical effort was subserved by a common network of areas, including the dorsomedial and dorsolateral prefrontal cortex, the intraparietal sulcus, and the anterior insula. Activity within these domain-general areas also covaried negatively with reward and positively with effort, suggesting an integration of these parameters within these areas. Additionally, the amygdala appeared to play a unique, domain-specific role in processing the value of rewards associated with cognitive effort. These results are the first to reveal the neurocomputational mechanisms underlying subjective cost–benefit valuation across different domains of effort and provide insight into the multidimensional nature of motivation.

## Abstract 2

Model-based fMRI in humans shows that cognitive and physical motivation are underpinned by overlapping neural substrates, but the amygdala plays a unique role in valuation of cognitive effort.

## Author summary

Rewards are rarely obtained without the motivation to exert effort. In humans, effort can be perceived in both the cognitive and physical domains, yet little is known about how the brain evaluates whether it is worth exerting different types of effort in return for rewards. In this study, we used functional magnetic resonance imaging (fMRI) to determine the neural and computational basis of effort processing. We developed two novel tasks that were either cognitively or physically effortful and had participants indicate their preference for a low-effort/low-reward versus a higher-effort/higher-reward version of each. Our results showed distinct patterns of reward devaluation across the different domains of effort. Furthermore, regardless of the type of effort involved, motivation was subserved by a large network of overlapping brain areas across the parieto-prefrontal cortex and insula. However, we also found that the amygdala plays a unique role in motivating cognitively—but not physically—effortful behaviours. These data impact current neuroeconomic theories of value-based decision making by revealing the neurocomputational signatures that underlie the variability in individuals’ motivation to exert different types of effort in return for reward.

## Introduction

Neuroeconomic theories highlight that a key component of motivation is evaluating whether potential rewards are worth the amount of effort required to obtain them [1, 2]. Behaviours are executed if they have sufficient “subjective value” (SV), which is based on how much a potential reward is discounted—or devalued—by the effort required to obtain that outcome [3]. A characteristic of these cost–benefit valuations is that they are inherently highly subjective and thus vary across individuals [4–6]. Some people are willing to invest a quantum of effort for a reward that others would not. However, not all types of effort are subjectively evaluated in the same manner. Some individuals may be willing to overcome physically demanding challenges but be averse to mental effort, while others might show the opposite profile. Understanding the mechanisms that underlie cost–benefit valuations across different domains of effort is crucial to understanding the variability in people’s motivation [7, 8], but little is known of the neural or computational basis of these mechanisms.

Current theories of value-processing suggest that the computation of SV occurs in a common, domain-general network of brain regions [9]. Single-cell and neuroimaging studies have implicated areas within the basal ganglia and parieto-prefrontal cortices in the computation of SV for rewards that are devalued by costs such as risk, delays, or probability [9, 10]. Separately, research on effort-based decision making has implicated areas including the anterior cingulate cortex (ACC) (area 32’), dorsolateral prefrontal cortex (dlPFC) (areas 8/9), anterior insula (AI), intraparietal cortex (area 7), and several amygdala nuclei [11–16]. However, critical unanswered questions are whether these effort-sensitive areas compute the subjective value of discounted rewards associated with effort costs and whether these areas are differentially sensitive to the nature of those costs.

To date, most research on effort-based decision making has either focused on the cognitive or physical domains in isolation [4, 17–19]. The only previous study to have examined the neural mechanisms associated with different types of effort required participants to *perform* a cognitively or physically demanding task [20]. Importantly, however, participants in that study were not engaged in the *choice* of whether it was worthwhile to invest effort for reward. Thus, although this study was useful in examining how the brain motivates the exertion of different effort costs, the neural substrates that underlie the subjective valuation of reward—and the decision of whether to engage in an effortful action—remain unknown. Increasingly, these decision processes are being recognised as a critical component of motivated behaviour, with evidence that aberrant effort-based decision making may be a key element of motivational disorders such as apathy [18, 19, 21].

Here, we used the computation of SV as a key operation to understand cost–benefit decision making across the domains of cognitive and physical effort [9, 22–24]. In contrast to classical accounts, recent research in animals suggests that the mechanisms that underpin cognitive and physical effort discounting might be separable. For example, animal studies of the amygdala have causally linked it to motivation and the devaluation of reward by effort costs [25, 26]. Recently, however, a novel rodent decision-making task showed dissociable effects of amygdala and frontal lesions on cognitive effort–based decisions [27]. Specifically, amygdala and ACC inactivations caused changes to behaviour during a cognitive effort task [27] that were different to those in physical effort tasks [2, 25, 26]. Furthermore, amygdala inactivation influenced individual animals differently, suggesting that the amygdala may play a distinct role in subjectively valuing rewards associated with cognitive effort. Such findings suggest that the computation of SV in the context of effort may not be within a domain-general network of valuation areas, as is often argued [9].

To establish whether the SV of different effort costs are processed within domain-general or domain-specific brain systems, the current study directly examined whether the neural mechanisms underlying subjective reward valuation are sensitive to different types of effort. We first trained participants on two tasks that were closely matched on many properties that are known to influence the valuation of a reward (e.g., probability, duration prior to outcome) [28] but differed in whether cognitive or physical effort was required to obtain rewards. In each, we parametrically varied effort in one domain while holding the demands of the other constant. Then, while being scanned with functional magnetic resonance imaging (fMRI), participants chose between a fixed low-effort/low-reward “baseline” option and a variable higher-effort/higher-reward “offer.”

Central to our paradigm was the use of computational models to calculate the SV of each effort and reward combination relative to the baseline option for individual subjects, which allowed us to calculate subject-specific discounting parameters for each of the cognitive and physical effort tasks. Using model-based fMRI, we then identified regions in which blood oxygen level–dependent (BOLD) activity correlated with these parameters. This revealed that cognitive and physical effort discounting occurred in largely overlapping neural areas, but in addition, the right amygdala contributed uniquely to cognitive effort valuation.

## Results

### Behavioural results

**Fig 1:**
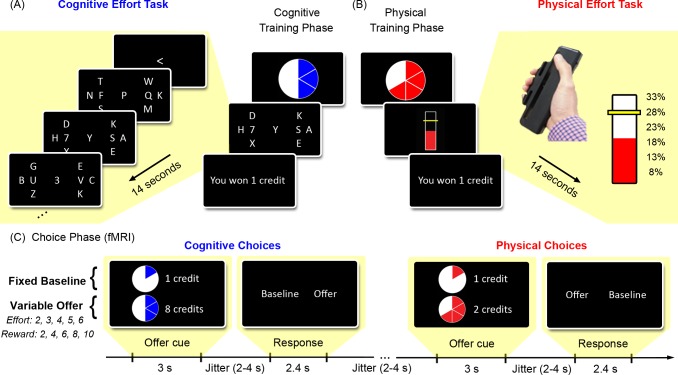
Cognitive and physical effort tasks. *Upper Panel*. Participants were first trained on the cognitive and physical tasks. Each trial commenced with a pie chart indicating the upcoming effort level. (A) The cognitive effort task utilised an RSVP paradigm. The main task was to detect a target “7” in one of two letter streams at either side of fixation (here initially indicated by “F” and “Q”). Each target stream was surrounded by three distractor streams. An arrowhead at the beginning of each trial indicated the initial target stream. During the trial, a central “3” was a cue to switch attention to the opposite target stream. Cognitive effort was manipulated as the number of attentional switches per trial (1–6). (B) The physical effort task required participants to maintain a sustained grip on a handheld dynamometer at one of six levels of force, as a function of their individually calibrated maximal voluntary contraction (MVC). *Lower Panel*. (C) After training, participants chose between a fixed low-effort/low-reward baseline and a variable high-effort/high-reward offer. Choices were made while being scanned with fMRI and were made separately for the cognitive and physical domains.

In the cognitive effort task [4], we employed a rapid serial visual presentation (RSVP) paradigm [29], in which participants fixated centrally while monitoring one of two target streams to the left and right of fixation for a target number “7” (Fig 1A). Each target stream was surrounded by three distractor streams. The target stream to be monitored was indicated at the beginning of the trial by a central arrow and, during the trial, participants had to simultaneously monitor the central stream for a number “3,” which would be a cue to switch their attention to the opposite target stream. We parametrically varied the amount of cognitive effort over six levels by increasing the number of times attention had to be switched between streams from one to six. We previously confirmed that this task was able to manipulate perceived cognitive effort while controlling for physical demands and reinforcement rates [4].

In the physical effort task, participants exerted one of six different levels of force on a handheld dynamometer (Fig 1B). The effort levels for each participant were defined as proportions of their individually calibrated maximum voluntary contraction (MVC) (8%, 13%, 18%, 23%, 28%, and 33%), as determined at the beginning of the experiment. The duration of each of the cognitive and physical effort trials was identical (14 s), ensuring that participants’ choices were not due to temporal discounting [30, 31].

Participants were first trained on each of the cognitive and physical effort tasks outside the scanner in counterbalanced order. They undertook an extensive training session of 60 trials for each task to familiarise themselves with the effort associated with each level in each domain and so that we could estimate performance measures for each task (see Materials and Methods). Participants were told that their reimbursement at the end of the study would be contingent on performance and that for each trial that they performed well, they would be awarded one credit, which would be later converted into a monetary amount. This training resulted in participants being rewarded on over 80% of trials, and a repeated-measures ANOVA revealed that, although there was a significant effect of effort (*F*(1.7, 57.2) = 7.48, *p* < .005), neither the main effect of domain nor its interaction with effort were significant (*p* > .05; S1 Fig). Importantly, this indicates that the reinforcement rates did not differ between tasks and ensured that subsequent effort-based decisions in the two domains could not be confounded by participants’ belief that they would be differentially successful at obtaining rewards across the two tasks.

The critical choice phase occurred after the training phase, while participants were being scanned with fMRI (Fig 1C). During this phase, participants made cost–benefit decisions for the cognitive and physical effort tasks separately. On each trial, they were presented with a fixed low-effort/low-reward “baseline” option and a variable high-effort/high-reward “offer.” The baseline option was an opportunity to perform the lowest level of effort for one credit, while the offer presented a higher number of credits (2, 4, 6, 8, or 10 credits) for having to invest a greater amount of effort (levels 2–5). Importantly, by providing participants with the identical range of reward options for both cognitive and physical effort, we could disentangle how cognitive and physical effort differentially devalued the identical rewards. In addition, in order to eliminate the effect of fatigue on participants’ decisions, they were not required to execute their choices within the scanner. Instead, they were instructed that they would be required to perform a random selection of ten of their choices at the conclusion of the experiment and that their remuneration would be based on these randomly selected trials.

Because separate decisions were made for the cognitive and physical tasks, we were able to estimate the extent to which the same amount of reward was devalued within each domain for each participant. An important feature of our design was that we temporally separated the presentation of the offer from that of the response cue. Thus, participants did not know which button corresponded to the baseline or offer until the onset of the response prompt. This ensured that we could examine activity time-locked to a cue from which SV would be processed independently, with activity related to these events not confounded by preparatory motor activity.

#### Effort sensitivity

**Fig 2:**
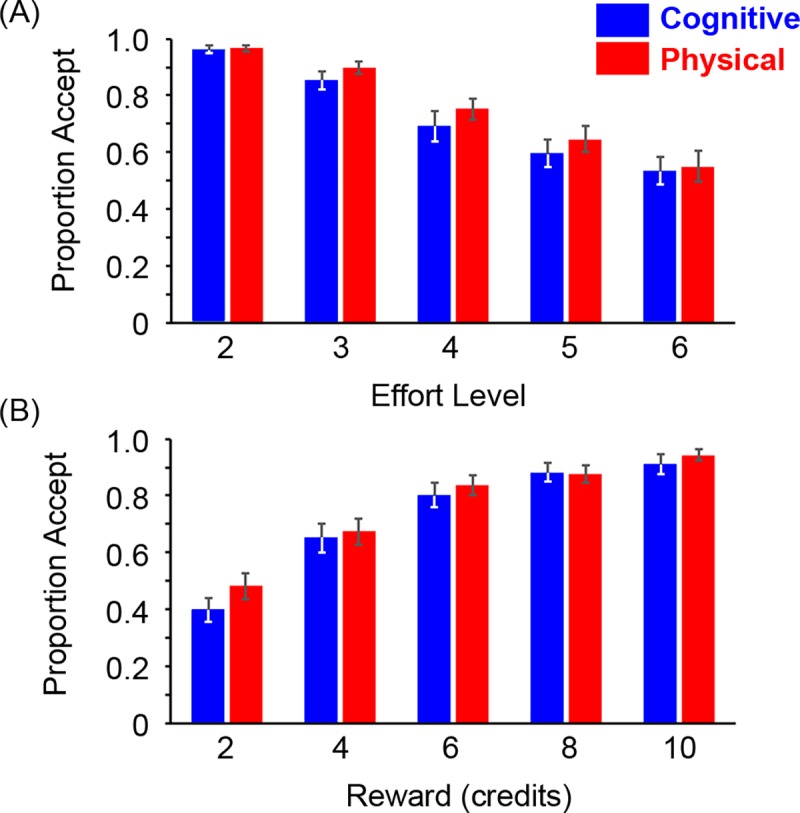
Group data reveal similar choice behaviour for cognitive and physical effort. Proportion of accepted offers (±1 standard error of the mean [SEM]) as a function of (A) effort and (B) reward for the cognitive and physical effort tasks. Underlying data for panels A–B can be found in S1 Data.

First, to probe for differences in effort sensitivity across the cognitive and physical domains, we examined the proportion of trials in which participants chose the more effortful offer, as a function of effort level (Fig 2A). A two-way repeated-measures ANOVA on the factors of domain (cognitive, physical) and effort (1–6) revealed a significant effect of effort, such that each level was different to every other level (*F*(2.05, 67.6) = 89.1, *p* < .001; all post-hoc comparisons *p* < .001, Bonferroni-corrected). However, neither the main effect of domain nor the two-way interaction was significant (domain, *F*(1, 33) = 0.82; interaction *F*(1.86, 61.4) = 0.60).

#### Reward sensitivity

We then performed the complementary analysis on reward sensitivity to analyse the proportion of offers chosen as a function of Reward (Fig 2B). Here, we found a significant main effect of Reward (*F*(2.04, 67.3) = 93.1, *p* < .001), such that each level was significantly different to every other level (all *p*-values < .05). However, neither the main effect of domain nor its interaction was significant (domain, *F*(1, 33) = 0.82; interaction, *F*(2.09, 68.9) = 1.42). Together, these results suggest that, at a group level, choice behaviour in the cognitive and physical effort tasks was indistinguishable.

#### Choice reaction time

Choice reaction time has previously been taken as an index of decision difficulty [32]. In order to ensure that cognitive and physical effort–based decisions did not differ in terms of decision difficulty, we analysed the median reaction times for participants to provide a response following the onset of the response prompt (S2 Fig). A repeated-measures ANOVA was conducted on the factors of domain (cognitive, physical) and effort (Levels 2–6), which showed no significant main effects or interactions (domain, *F*(1, 33) = 0.59; effort, *F*(4, 132) = 2.31; domain x effort, *F*(4, 132) = 2.06); all *p*-values > .05). The analogous domain x reward ANOVA also failed to reveal any significant results (domain, *F*(1, 33) = 0.34; reward, *F*(4, 132) = 1.64; domain x reward, *F*(4, 132) = 1.65; all *p*-values > .05). This verifies that the cognitive and physical decision-making tasks were similar in terms of times needed to make a choice.

#### Logistic regression of choice behaviour

Next, we sought to exclude probability discounting as an explanation for participants’ choices (S3 Fig). We performed a logistic regression using a regressor that takes the proportion of rewards obtained at each level of effort in the training session as predictors of choice during the fMRI task. This can be considered a proxy for a participant’s prediction of the probability that they would be rewarded at each level. For each domain, effort, reward, and reinforcement rates (i.e., the proportion of trials rewarded at each effort level in the training session) were entered into the model and allowed to explain shared variance. Thus, we were able to examine whether any of the parameters could explain behaviour significantly over and above any correlations with other variables. We entered reinforcement, reward, and effort as covariates in a separate general linear model for each participant and for each effort domain. Beta values were then normalised to *t*-statistics as *β*/SE(*β*) to compensate for the possibility of poor estimates of *β*s in participants with low levels of variance for any variable. Finally, we ran nonparametric Wilcoxon signed-rank tests on each of the covariates against zero.

These analyses revealed that effort scores significantly predicted choice behaviour in the negative direction (i.e., higher effort levels were chosen less frequently; *t*(cog) = –1.92, *Z* = –4.69, *p* < .0001; *t*(phys) = –1.86, *Z* = –4.59, *p* < .0001), and that reward scores significantly predicted choice behaviour in the positive direction (i.e., higher rewards were chosen more frequently; *t*(cog) = 1.90, *Z* = 4.88, *p* < .0001; *t*(phys) = 1.81, *Z* = 4.78, *p* < .0001). Importantly, however, reinforcement rates did not significantly predict choice behaviour across the group (*t*(cog) = 0.22, *Z* = 0.54, *p* = .28; *t*(phys) = 0.10, *Z* = –0.08, *p* = .94). These analyses therefore demonstrate that (1) effort and reward each predict choice behaviour and (2) reinforcement rates in the training phase did not affect participants’ preferences, which excludes probability discounting as an explanation for choice behaviour in our study.

#### Computational modelling of choice behaviour

Despite the group data showing no apparent differences in behaviour across domains, such an approach collapses across the individual differences inherent in this paradigm, in which some participants may differentially prefer effort in one domain over another (see, for example, S4 Fig). Several previous studies have attempted to capture the pattern of such individual differences by computationally modelling subjective devaluation of reward by effort [5, 14, 33]. Based on this literature, we fitted linear, hyperbolic, parabolic, and exponential functions to participants’ choices in the cognitive and physical effort tasks using a *softmax* function and maximum likelihood estimation (see Materials and Methods).

**Fig 3:**
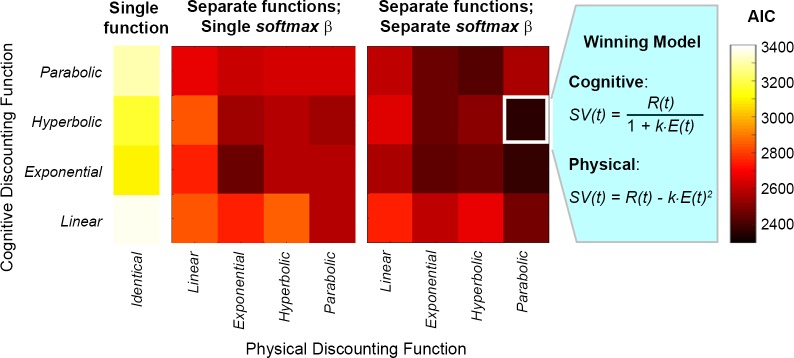
Computational modelling revealed that reward devaluation by cognitive and physical effort was best described by different functions. The winning model indicated that cognitive valuation was best fitted by a hyperbolic function and physical valuation by a parabolic function. Three classes of model were compared, based on whether cognitive and physical effort were assumed to discount reward with single or separate functions, or single or separate *softmax β*s. Models were fitted using maximum likelihood estimation and compared with an Akaike Information Criterion (AIC) (illustrated here). Additional model comparisons using a Bayesian Information Criterion (BIC) revealed the identical pattern of results (S5 Fig). *SV*(*t*) represents the subjective value of the offer on trial *t*, *R* is the reward in credits (2, 4, 6, 8, or 10), *E* is the effort level (0.2, 0.4, 0.6, 0.8, or 1.0), and *k* is a subject-specific discounting parameter. Underlying data can be found in S1 Data.

We created three separate classes of model (Fig 3; S5 Fig). These classes assumed the following: (1) the same discounting and *softmax* parameters for choices across both tasks (i.e., equal motivation and equal stochasticity when choosing to exert cognitive or physical effort), (2) the same discounting parameter but distinct *softmax* parameters across both tasks (equal motivation but distinct stochasticity across domains), or (3) separate discounting and *softmax* parameters for both tasks (different levels of motivation and different levels of stochasticity). In total, we compared 36 different models to examine whether rewards were valued differently at an individual subject level when associated with different effort costs and also the nature of the function that characterised the valuation in each domain. Model fits were compared with the Akaike Information Criterion (AIC) (Fig 3) [34] and Bayesian Information Criterion (BIC) (S5 Fig) [35], both of which converged on identical results.

The winning model showed that participants’ choice responses were best described by separate discounting parameters (*k*) and separate *softmax β*s for the cognitive and physical tasks. Specifically, the cognitive task was best modelled with a hyperbolic function and the physical task with a parabolic function:

Cognitive effort:             $SV(t)=R(t)∙\frac{1}{1+k∙E(t)}$
Physical effort:                 *SV*(*t*) = *R*(*t*)−*k*∙*E*(*t*)^2^

in which S*V*(*t*) represents the subjective value of the offer on trial *t*, *R* is the reward in credits, *E* is the effort level (0.2, 0.4, 0.6, 0.8, or 1.0), and *k* is a subject-specific discounting parameter, which describes the gradient of each individual’s discounting function (the higher the *k* value, the steeper the discount function). Note that each individual’s discounting function is referenced to the SV of the baseline offer (one).

This model highlights that participants valued rewards differently, both in terms of the extent to which they valued the reward and the mathematical nature of the discounting effect, depending on the effort domain with which they were associated. This is particularly striking given the fact that the rewards associated with the different effort costs were matched. There was no significant correlation between the discounting parameters for cognitive and physical effort (*ρ* = 0.22, *p* = .20). In addition, there was no significant difference between the *softmax β*s for the cognitive and physical tasks (*β*_cog_ = 10.29 [SE 3.87]; *β*_phys_ = 6.82 [SE 3.04]; *t*(33) = 0.84; not significant), which indicates that there were no systematic differences in participants’ consistency when making choices in the two domains.

#### Self-reported effort

For both the cognitive and physical effort tasks, we used the mental and physical effort subscales of the NASA Task Load Index to confirm that each task was perceived as significantly more effortful in the corresponding domain and that participants were able to subjectively perceive the increments in effort for each task (Materials and Methods) [36]. Overall, we found that subjective mental or physical demand ratings were always higher in the corresponding task and that this difference increased with effort (S6 Fig). This confirms that each task was able to differentially modulate behaviour in the corresponding domain.

### Imaging results

Using the modelling parameters derived above, we computed the SV for the effort and reward combinations on every trial and used the difference in value between the SV of the chosen offer and the value of the baseline as a parametric regressor modelled to the onset of the offer cue [37]. Many studies have shown that regions we hypothesised would be engaged by cost–benefit valuations are sensitive to the difference in the SV of two options rather than to the SV of an offer per se [31, 37]. Thus, we fitted the SV difference on each trial within the cognitive domain as a parametric modulator time-locked to the onset of each cognitive offer and performed the corresponding analysis for offers in the physical domain. This allowed us to examine activity covarying with SV for the cognitive and physical domains separately. These parametric modulators were defined based on the discounting parameters estimated for each participant’s choice behaviour. We considered significant those voxels which survived whole brain–level, voxel-wise corrections for multiple comparisons (*p* < 0.05, corrected for family-wise error [FWE]).

#### Subjective valuation of cognitive effort

First, we conducted a *t*-test to identify areas in which activity parametrically varied with SV difference between the offer and the baseline option for the cognitive task (S7 Fig). In line with our hypotheses, we found a focus of activity in the dorsal anterior cingulate cortex (dACC), spanning the dorsal part of the caudal cingulate zone, posterior to the genu of the corpus callosum, and crossing the cingulate sulcus (Brodmann area [BA] 24c’) into the adjacent part of the dorsomedial prefrontal cortex (dmPFC) (BA 8). Further foci were identified in the middle frontal gyri of the dlPFC (area BA 9/46), extending into the inferior frontal sulcus (IFS) bilaterally (IFS, BA 44), the bilateral intraparietal sulcus and adjacent superior and inferior parietal lobules (BA 7, 39), and bilateral portions of the anterior, dysgranular insula (Table 1).

**Table 1:** Areas sensitive to the SV difference between the chosen option and baseline for (A) cognitive effort, (B) physical effort, and (C) the conjunction of both domains. Clusters are significant at a voxel-wise threshold of *p* < .05, corrected for family-wise error. Coordinates are given in Montreal Neurological Institute (MNI) space. aIPS, anterior intraparietal sulcus; pIPS, posterior intraparietal sulcus; dACC, dorsal anterior cingulate cortex; dmPFC, dorsomedial prefrontal cortex; dlPFC, dorsolateral prefrontal cortex; MFG, middle frontal gyrus; SFG, superior frontal gyrus; IFS, inferior frontal sulcus.

Area	Peak voxel	*k*	*Z* value	Voxel *p* (FWE)
**Cognitive Effort**				
Right IPS	36, –44, 38	1,137	6.62	< .001
dACC/dmPFC	–4, 22, 44	507	6.55	< .001
Left IPS	24, –60, 42	1,467	6.48	< .001
Right insula	34, 22, 2	86	5.89	< .001
Left dlPFC (IFS)	–44, 26, 26	58	5.39	< .001
Right dlPFC (IFS)	44, 36, 30	82	5.29	< .001
Left insula	–28, 22, 4	29	5.28	< .001
**Physical Effort**				
Right IPS	32, –68, 36	760	5.70	< .001
dACC/dmPFC	10, 22, 42	317	5.69	< .001
Right dlPFC (IFS)	44, 32, 28	90	5.39	< .001
Right dlPFC (SFG)	26, 20, 60	13	4.92	< .001
**Conjunction of Cognitive and Physical Effort**				
dACC/dmPFC	–4, 20, 46	578	6.61	< .001
Right IPS	24, –60, 40	1,267	6.29	< .001
Right dlPFC (MFG, IFS)	44, 32, 36	145	5.83	< .001
Right insula	34, 22, 0	77	5.62	.001
Left pIPS	–26, –64, 40	43	5.09	.009
Left aIPS	–38, –42, 40	42	5.08	.01
Left IFS	–44, 28, 26	6	4.89	.023

#### Subjective valuation of physical effort

We then performed the corresponding analysis for the physical task by conducting a *t*-test for activity that parametrically varied with SV difference between the offer and baseline, time-locked to the presentation of these options (S7 Fig). At FWE-corrected thresholds of .05, this analysis revealed activity in a largely similar network of areas as for cognitive effort valuation, including dorsal ACC/dmPFC (area 24c’/area 8); right superior and middle frontal gyri (9/46) and adjacent inferior frontal sulcus; and the right intraparietal sulcus (IPS) (Table 1).

#### Domain-general subjective valuation

**Fig 4:**
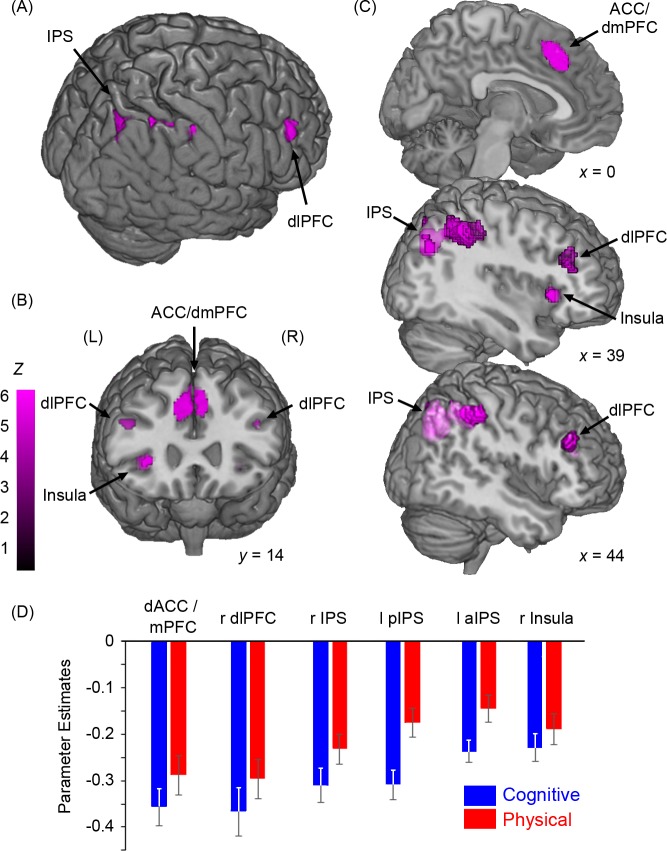
A network of domain-general areas was involved in the valuation of cognitive and physical effort. Conjunction analyses demonstrated that the activity of these areas covaried with SV parameters, independent of the type of effort cost. (A) Whole-brain render, showing foci in the right intraparietal sulcus (IPS) and the right dorsolateral prefrontal cortex (dlPFC), comprising the middle frontal gyrus (MFG) and adjacent inferior frontal sulcus (IFS). (B) Coronal section showing activity in the anterior cingulate cortex (ACC) and adjacent dorsomedial prefrontal cortex (dmPFC), IFS, and right insula. (C) Sagittal sections showing ACC/dmPFC, IPS, and dlPFC activity. (D) Parameter estimates for domain-general clusters. Underlying data for panel D can be found in S1 Data.

To address the critical question of whether areas exist that estimate SV independent of domain, we first asked which of the areas identified above for SV in the cognitive and physical domains overlapped. This analysis showed a network of areas that appear to subserve subjective valuation in both domains, specifically in the dACC/dmPFC (area 24c’/area 8), the right dlPFC (9/46), the IPS, and the right anterior insula (S7 Fig). To statistically confirm that these areas were implicated in domain-independent subjective valuation, we performed a conjunction analysis on a factorial ANOVA for the cognitive and physical tasks, with a whole-brain, FWE-corrected *p*-value of .05 [38]. Importantly, all of the areas identified in the overlap were significantly active for the conjunction analysis at this threshold (Fig 4, Table 1). Furthermore, activity in many of these regions varied positively with the effort level of the offer and negatively with reward level (S8 Fig). Interestingly, although the ventral striatum (VS) and ventromedial prefrontal cortex (vmPFC) have been previously implicated in value-based decisions, we did not detect any statistically significant clusters of activity within these regions, even when they were specifically probed using regions of interest (ROIs) based on recent studies [20, 39] (see Materials and Methods).

#### Domain-specific subjective valuation

To determine whether any regions encoded value difference significantly greater for one domain relative to the other, we performed *t*-tests on value difference between the cognitive and physical tasks. Considerable evidence links the basolateral amygdala to motivation, and it plays an especially critical role in effort-based decisions [26, 40–42]. Coupled with recent findings implicating the amygdala as being differentially sensitive to cognitive and physical effort discounting [27], we used the amygdala as an a priori region of interest for these analyses and tested the hypothesis that this region would code for cognitive SV but not physical SV. We therefore conducted *t*-tests at a whole-brain, uncorrected statistical threshold of *p* < .001, with a small volume correction using an amygdala mask from the Harvard-Oxford Subcortical Structural Atlas provided by the Harvard Centre for Morphometric Analysis (http://www.cma.mgh.harvard.edu/fsl_atlas).

**Fig 5:**
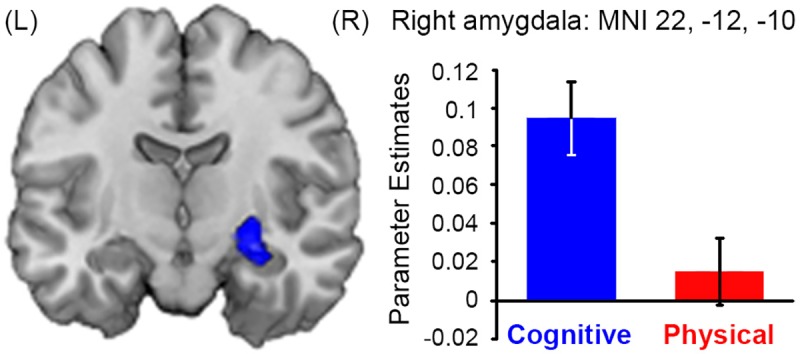
The right amygdala uniquely tracked the SV of rewards associated with cognitive costs. Coronal section showing activity within the right amygdala that parametrically varied with SV of the cognitive (but not physical) offer. Underlying data be found in S1 Data.

Crucially, the contrast of cognitive effort > physical effort revealed a significant cluster in the right amygdala (MNI 22, –12, –10; *Z* = 4.11; cluster *p*_FWE_ = .04; Fig 5). In contrast, the reverse comparison of physical > cognitive revealed no suprathreshold activity in the amygdala nor in any other region of the brain at a whole-brain corrected threshold. Finally, ROIs centred on the VS and vmPFC did not reveal any evidence of domain-specific activity for either the cognitive > physical or physical > cognitive contrasts.

Together, these results implicate a network of domain-general areas that encode the subjective value of both cognitive and physical effort and that also encode the raw value of the effort and reward on offer. These data also suggest a unique contribution of the right amygdala to encoding subjective value in the cognitive domain.

#### Control fMRI analyses for difficulty, risk, and generalisability

To ensure that all of the reported activations were due to subjective valuation rather than decision difficulty, we performed two control analyses by computing separate indices of perceived difficulty (S9 Fig)—namely, decision response time and a measure derived from our modelling analysis (-|*p*-0.5|, in which *p* represents the choice probabilities derived from the *softmax* equation from the winning model; see Materials and Methods). We used each of these variables as parametric regressors again time-locked to the onset of the offer cue. Importantly, neither the amygdala nor any of the areas identified as being active for the conjunction of cognitive and physical effort–based decisions signalled choice difficulty for any of these measures (Materials and Methods; S9 Fig).

In addition, although the logistic regression described above confirmed that performance at each effort level in the training session could not account for choice behaviour in the scanner, we wished to confirm that any perceived risks associated with each effort level did not contribute to the activity we found for areas involved in subjective valuation (S10 Fig). We therefore performed three separate control analyses to check for this. In one analysis, we took the error rates for each effort level in the training session and used these as parametric regressors time-locked to the offer cue. In the remaining two analyses, we used performance measures in the cognitive effort task (*d’*, i.e., *Z*(Hits)–*Z*(False Alarms)) and the physical effort task (total time within the force window required for reward) as regressors. In none of these three analyses did the areas signalling subjective value reveal any activity for perceived risk associated with each effort level (Materials and Methods).

Finally, we note that these findings occurred in the context of two tasks that were best described by separate effort discounting functions (hyperbolic and parabolic). Could the neuroimaging findings simply be due to these specific functions characterising the valuation, or could the domain-general and domain-specific regions identified here generalise to other effort-based tasks described by different functions? To speculate on this question, we conducted a series of exploratory analyses using only a single function to model effort discounting across both domains (Materials and Methods). This analysis revealed a pattern of domain-general and domain-specific effects that was largely similar to the present study, albeit at a reduced statistical threshold. This suggests that the present findings are unlikely to be due to idiosyncratic features of the hyperbolic and parabolic functions described in the winning model.

Together, these results show that the dACC/mPFC, dlPFC, insula, and IPS integrate reward and effort information to process SV in a domain-independent manner and that the amygdala appears to process SV specifically related to cognitive effort. Importantly, all of these activations are independent of choice difficulty or perceived risk. Furthermore, the domain-general and domain-specific effects are unlikely to be driven by the specific nature of the discounting functions described by our winning model.

## Discussion

We used model-based fMRI to determine whether shared or separate neurocomputational mechanisms underlie cost–benefit valuation in the cognitive and physical domains. Computational modelling revealed that individuals were differentially sensitive to cognitive and physical effort. Neuroimaging data showed that activity in several areas previously implicated in effort processing covaried with the subjective value of rewards independent of effort domain. This included the dACC, dmPFC, dlPFC, IPS, and anterior insula. Importantly, activity within many of these areas also covaried with absolute reward and effort levels, suggesting an integration of these parameters within these areas. However, in contrast to the view that SV is processed in an entirely domain-general manner, an ROI analysis revealed that the right amygdala appeared to process SV uniquely for rewards associated with cognitive and not physical costs. Importantly, none of these results could be explained by choice difficulty or perceived risk. Together, these data indicate that cost–benefit valuation in the human brain is underpinned mostly by a common, domain-independent mechanism but that the amygdala may play an important role in valuing rewards associated with cognitive effort. These results therefore suggest that the classical view of a domain-general set of brain regions for valuation cannot fully account for the subjective valuation of rewards associated with all effort costs [9].

To our knowledge, no study to date has examined the neural correlates of SV associated with cognitive versus physical effort in a single paradigm. The only study that has addressed the nature of cognitive and physical effort examined the processing of raw magnitudes of effort and reward without considering individuals’ subjective valuations and did not require subjects to make choices about whether the effort was worth exerting to obtain the reward [20]. Such an approach is common in the literature and assumes that rewards have a similar effect across individuals to exert the associated effort [11, 13, 43, 44]. However, preferences vary depending on subject-specific cost–benefit valuations, and SVs potentially afford a more sensitive measure of capturing individual differences in motivation [9, 22, 23, 45]. Furthermore, SV has been proposed as an important entity in understanding apathy in healthy individuals as well as those with clinical disorders of motivation [8, 21, 46]. Defining the neural and computational mechanisms that underlie the choice to exert effort for reward is therefore crucial to understanding the variability in motivated behaviour across individuals. In the present study, by parametrically varying effort across six levels in both domains, we were able to computationally model SVs for individual participants and therefore more closely examine the key computations that underpin choice behaviour and motivation.

Our paradigm had several other advantages. First, the protocol involved manipulating effort in two separate domain-specific tasks, as opposed to requiring participants to exert a combination of both forms of effort to attain specific rewards in each trial [20]. We were therefore able to examine choice behaviour for identical rewards in each domain independently. Second, although many studies have examined the processing of effort and reward, the majority may have been confounded by motor execution for the choices or preparatory activity related to an upcoming effortful exertion. In the design used here, it was possible to investigate activity specifically related to decisions based on SV by temporally separating the choice process from the preparation or execution of the effortful act. Third, by controlling the temporal parameters of both the cognitive and physical effort tasks, it was possible to eliminate delay discounting as an explanation of choice behaviour [5, 47, 48]. Fourth, by using computational modelling approaches, we were able to examine activity that varied with SV. Finally, by ensuring that reinforcement rates were similar for the six levels of effort within and across domains, it was possible to ensure that probability discounting could not have contributed to our findings. Thus, the study reported here isolates the effect of SV on choice and motivation independently of many effects that can confound studies examining effort-based decision making. As such, we can effectively rule out the possibility that several regions that we identified were only related to the energisation of behaviour and not to motivation or the valuation of behaviour [49].

Our model comparisons indicated that individuals valued rewards differently when associated with cognitive and physical effort. This was demonstrated by the winning model, which specified separate discounting functions requiring separate discounting parameters for cognitive and physical effort. This conclusion was also supported by the more general pattern of the computational modelling results, which showed that the models assuming equal reward devaluation across cognitive and physical effort (i.e., those assuming a single discounting parameter) provided poorer fits than those that assumed separate discounting parameters. This finding that different functions best fitted cost–benefit valuations for cognitive and physical effort most likely reflects differential sensitivities to effort in the two domains. Our finding that a parabolic function best accounts for participants’ choice behaviour in the physical effort task is in keeping with previous observations [33]. In contrast, effort discounting in the cognitive domain has been much less studied [6], and it is likely that the specific shape of a discounting function will depend on the specific cognitive faculty being tested (e.g., attention versus working memory). However, the key point for the present study is that, in the tasks that we used, identical rewards were valued distinctly across both domains.

Strikingly, despite rewards being devalued at different rates and in a mathematically distinct manner across the two domains, a largely overlapping network of regions was involved in processing the SV of rewards devalued by both the cognitive and physical effort cost. It is important to note that this finding does not rely on the generalisability of these specific functions to other cognitive or physical effort–based tasks. However, the fact that effort discounting in our task is best described by separate functions does considerably strengthen this result, as it implies that any differences between cognitive and physical effort cannot simply be a matter of scale (e.g., some participants finding one task more effortful than the other). Rather, it suggests a possible difference in the underlying mechanism between the two processes. Furthermore, the separate discounting functions render the imaging results more compelling by showing that the SVs computed from entirely different functions nevertheless engage overlapping brain regions.

Regardless, a question that remains is whether the same pattern of results would be achieved in a cognitive and physical effort task that were best described by the identical discounting function. Exploratory analyses using a single function to model choice across both domains revealed a pattern of domain-general and domain-specific effects that were essentially similar to those of the primary analyses. However, it remains for future studies to verify the conclusions from our study in the case of cognitive and physical effort tasks that are best described by identical discounting functions.

Interestingly, most of the domain-general areas that encoded subjective value also showed a significant negative effect of reward and a significant positive effect of effort. The findings that many domain-general areas that encode SV also encode raw reward and effort levels are not incompatible—indeed, one interpretation is that these regions integrate the reward and effort on offer into a value signal. Although many previous studies have examined the neural basis of processing SV [9], we believe this is one of the first demonstrations that regions of the brain can process a SV formed from costs that devalue rewards at different rates. Furthermore, although some of these domain-general regions may be involved in processing decision difficulty in certain contexts [50], this is not always the case [51], and none of the regions identified in the present study were found to encode choice difficulty across both the cognitive and physical domains.

The key to elucidating the neural basis of cost–benefit decision making will be understanding how this domain-general network learns or forms a valuation of rewards associated with different forms of effort [15, 22]. A central role of the dorsal ACC/dmPFC in value-based decision making and motivation is considered by some to be in signalling the value of a behaviour in comparison to alternatives [52, 53]. The study reported here extends this notion by showing that this region not only processes the SV of an offer but also integrates effort and reward information independent of the nature of the effort cost [50, 52, 54]. In addition, single-unit studies have shown that dACC/dmPFC neurons signal the net value of rewards associated with effort, and the necessity of this region in cost–benefit valuation has been demonstrated by lesion studies that report that inactivation of medial prefrontal cortex impairs an animal’s ability to overcome effort costs [15, 16, 25, 48, 55]. Recently, several human studies have also shown this region to be important in calculating choice value for effortful rewards. Although the majority of these have been in the physical domain [11, 13, 14], a recent investigation reported a similar pattern for cognitive effort [56].

Neurons sensitive to reward information have been identified in the dlPFC [55, 57–59], and the activity of lateral prefrontal areas in humans correlates with predicted SVs that guide decision making [60]. Lateral intraparietal neurons have been found to signal expected value [61], and parietal activity has been reported in tasks requiring value comparisons [62, 63]. Lastly, insular activity is negatively correlated with the SV of rewards associated with higher effort [14, 64], and dopaminergic responses, which play an important role in motivated decision making, exhibit greater variability in the insula with less willingness to expend effort for reward [65]. Our findings extend this body of data by showing that the process of subjective reward valuation occurs independent of the nature of effort costs, and suggest that it is underpinned by activity in a the dACC/dmPFC, dlPFC, IPS, and anterior insula.

Do these regions of domain-independent areas comprise a network for subjective valuation? Tracer studies in macaque monkeys and neuroimaging studies in humans suggest that these domain-independent regions are monosynaptically connected. The upper bank of the dorsal anterior cingulate sulcus is connected to the anterior portions of the insula, several amygdala nuclei, and BA 9/46 in the lateral prefrontal cortex. Similar projections exist between each of these locations and the other domain-independent regions within this putative network [42, 66–69]. In addition to the connectional anatomy, it has been noted that these same domain-independent regions are activated during a variety of different cognitive and motor control tasks [70, 71]. It has been argued that this multiple-demand (MD) network is involved in flexibly controlling the cognitive processes required across a large number of tasks [70].

In this context, our results could be taken as support for the notion that this network is activated independent of the nature of the cost or associated behavioural domain. However, our findings also suggest a more nuanced interpretation of the functional properties of the MD network. In our study, activity in this network was influenced by the value of working and not by the demand alone. Moreover, as highlighted above, these areas contain single neurons that respond to reward valuations, and the BOLD signal in these regions has been shown to scale with subjective reward valuations in studies investigating temporal discounting or probabilistic reward-based decisions. Thus, a more refined account might be that the MD network is crucial for motivating behaviours across different domains of behaviour. Such a notion would explain why these regions are activated during many cognitive and motor tasks in which motivation must be sustained for successful performance [72].

Importantly, we found evidence of domain specificity for cognitive effort valuation, specifically in the right amygdala. The amygdala is known to play an important role in reward valuation, and single-unit recordings have demonstrated that neurons here encode the value associated with individual items [26, 73–75]. Recent evidence points to the amygdala as playing a crucial role in effort-based decision making in rodents, with neurophysiological data showing that the amygdala plays an important role in valuing effort [40, 41]. Recently, some have proposed that the amygdala is sensitive to different types of effort costs [27] and also highlighted the key role for this region in the flexible control of cognitive processes. However, drawing a definitive conclusion, especially in humans, requires comparisons across species and across tasks.

Substantial differences exist between the paradigms used in valuation studies and include differences in reinforcement schedules, training intervals, reward magnitudes, and contrast effects. Furthermore, previous effort-based tasks have not tightly controlled the contributions from each domain to their manipulations of effort, thus making it difficult to compare the relative contributions of the two domains. Indeed, such discrepancies may even underlie varying amygdala involvement in cost–benefit decision-making tasks across cognitive and physical effort. In our study, we designed each of our closely matched tasks to hold all features constant except for the type of effort involved, which was maximised in each domain relative to the other. We were therefore able to provide more direct evidence that the human amygdala may be differentially involved in cognitive over physical effort valuation. Nevertheless, while our result is consistent with the preceding studies noting potentially dissociable roles of the basolateral amygdala for cognitive and physical effort–based decisions, the finding of amygdala domain specificity does deserve replication in future studies and would be even more compelling if it was demonstrable at a whole-brain level.

Interestingly, previous studies have shown that the VS and vmPFC are engaged when processing value [20, 39, 76]. Here, we found no such activity for either cognitive effort, physical effort, or the conjunction. This was the case even after specifically probing these areas with regions of interest defined on the basis of previous studies. A key difference between this study and all previous studies implicating the VS and vmPFC in value processing is that previous tasks required effort to be exerted while participants were being scanned, and most of the effects may have been related to the execution of the effortful task rather than to the choice of whether the effort was worth exerting. This may suggest that the VS and vmPFC process value primarily when value may guide or motivate the execution of a behaviour that will be followed immediately by a rewarding outcome, rather than in the evaluation of whether resources should be allocated to a task at all.

Rewards in real life are rarely obtained without effort. Our model-based fMRI approach revealed that effort discounting in the cognitive and physical domains is underpinned by largely shared neural substrates but that the amygdala uniquely contributes to cognitive effort valuation. Importantly, neither delay nor probability discounting can account for our results. It has been postulated that disorders of diminished motivation—such as apathy and abulia—which are manifest in multiple neurological and psychiatric conditions, may be characterised as a diminished willingness to exert effort for reward [46, 77]. Our findings may therefore help us understand the neural basis for such disorders of motivation by providing an insight into their multidimensional nature and identifying potential neural foci that might be manipulated to modulate motivation [78].

## Materials and methods

### Participants

This study was approved by the Central University Research Ethics Committee of the University of Oxford (MSD-IDREC-C1-2014-037). We recruited 38 young, healthy, right-handed participants. All participants had no history of neurological or psychiatric illness and were not taking regular medications. Four participants were excluded: 2 for failing to provide responses on a high proportion of trials while being scanned (over 9%), and a further 2 because of excessive head motion within the scanner (more than 5 mm of translation). The final group of 34 participants (23 females) had a mean age of 24 y (range 19–39).

### Method

All participants were behaviourally trained on a cognitively effortful task and a physically effortful task prior to being scanned. These extensive training sessions were aimed at familiarising participants with the effort associated with all levels for both tasks. The training phase for each task began with 18 practice trials (3 per effort level) and was followed by a further 60 trials to reinforce behaviour (10 per effort level). Behavioural analyses of task performance were conducted on the latter 60 trials. After training, we scanned participants while they made economic decisions based on how much effort they would be willing to trade off for varying levels of reward. The order of training in the physical and cognitive effort tasks was counterbalanced across participants.

#### Cognitive effort task

Cognitive effort was operationalized as the number of attentional switches that were performed in an RSVP paradigm [29]. This task was implemented in Presentation software (www.neurobs.com). The main task required participants to monitor one of two target letter streams at either side of fixation for a target number “7.” Each target letter stream was surrounded by three distractor letter streams.

The main task was to fixate centrally while monitoring two target letter streams to the left and right of fixation for the appearance of a target number “3.” There was a total of three targets per trial, and participants had to press the space bar on detecting each target. At the beginning of each trial, a left or right arrowhead appeared at fixation to indicate the target letter stream to which participants initially had to attend (left or right). Importantly, during the trial, they were required to switch their attention to the opposite target letter stream at the onset of a number “7” that appeared centrally. The number of attentional switches in each trial varied from 1 (effort level 1) to 6 (effort level 6), and these switches occurred at pseudorandom intervals during each trial. At the conclusion of each trial, participants would receive feedback with regards to the number of misses and false alarms on that trial. There were in total 40 serial stimulus presentations, each of which lasted 350 ms. Participants had to detect at least one of the three targets and commit no more than two false alarms to be rewarded with one credit.

During the training phase, participants were rewarded with one credit for each successfully completed trial and were told that the number of credits they accrued would be used to determine their remuneration. Feedback, in terms of whether the credit was successfully attained, was provided at the conclusion of each trial.

#### Physical effort task

Physical effort was operationalized as the amount of force that was exerted on a handheld dynamometer (SS25LA, BIOPAC Systems, United States). Participants were seated in front of a computer running Psychtoolbox (http://psychtoolbox.org) implemented in Matlab (Mathworks, US) and held the dynamometer in their dominant (right) hand. At the beginning of each session, the dynamometer was calibrated to each participant’s maximal voluntary contraction (MVC). Participants were instructed to squeeze the dynamometer as strongly as possible, and the maximum contraction reached over three trials was taken as each participant’s MVC.

The main task required participants to maintain a constant contraction at one of six levels of effort, which were defined as proportions of each participant’s previously determined MVC (8%, 13%, 18%, 23%, 28%, and 33%). Each trial was preceded by a cue indicating the level of effort that would be required. The cue took the form of a red pie chart, with the number of slices indicating the corresponding effort level (1–6). Participants were then presented with a vertical bar, which provided them with real-time feedback on their force as they attempted to achieve the target effort level, indicated by a yellow line superimposed on the bar. For each trial to have been considered successful, participants had to maintain their contraction within 2.5% of the target effort level for at least 50% of the 14-s trial duration. These force parameters were chosen following a series of pilot studies that showed them to be achievable by most participants. The trial duration was chosen to match that of the cognitive effort task, described below.

As in the cognitive effort paradigm, participants were again rewarded with one credit for each successfully completed trial and were provided with feedback regarding their success or failure at the end of each trial. All participants were reinforced in both the cognitive and physical effort tasks at rates of greater than 80%.

#### Choice period—within scanner

After being trained on the cognitive and physical tasks, participants were positioned in the fMRI scanner and performed an economic decision-making task. Participants were presented with two combinations of effort and reward and were instructed to choose the preferred option. One option was a high-effort/high-reward “offer,” which varied from effort level 2–6 and could be worth 2, 4, 6, 8, or 10 credits. This variable offer was always contrasted against a “baseline” option of the lowest level of effort (effort level 1) for the lowest reward (1 credit) within the same domain (cognitive or physical). Participants were instructed to choose either the “offer” or “baseline” option, depending on which was preferable to them. In order to eliminate the effect of fatigue, participants were only required to indicate their choices while being scanned and were told that they would be required to perform a random selection of ten of their chosen options from each of the cognitive and physical tasks at the conclusion of the experiment to determine their remuneration. All participants were paid £30 for their participation. However, participants were under belief that their payment and the effort they would have to exert to obtain rewards would depend on the choices they made inside the scanner.

Cognitive and physical effort trials were randomly intermixed. Each offer was presented for 3 s. The “offer” and “baseline” options were presented above or below fixation. The offer period was separated from the choice period by a random jitter of 2 to 4 s. During the choice period, the words “offer” or “baseline” appeared on the left or right side of the screen, and participants entered their preference by pressing the corresponding button on an MRI-compatible button box held in their right hand. The choice period lasted 2.4 s and was then separated from the next trial by a variable jitter of 2 to 4 s. In designing the fMRI task, we carefully checked the parametric regressors upon which we made inference for rank deficiency and ensured that all correlations between these regressors and any other had a correlation coefficient of less than 0.3.

#### fMRI scan acquisition

Functional MRI data were collected in a 3 Tesla Siemens Verio scanner at the Oxford Centre for Functional Magnetic Resonance Imaging of the Brain (FMRIB). The scanner was equipped with a four-channel Nova birdcage headcoil for signal transmission and reception. Stimuli were displayed on an MRI-compatible monitor positioned at the head of the scanner bore, which was visible to participants through a head coil–mounted mirror. Echo-planar images (EPIs) were acquired with a tilted-plane sequence with a pitch of 30° [79] using a gradient-echo pulse sequence and sequential slice acquisition (T_R_ 3000 ms; T_E_ 30 ms; flip angle 87°; 48 contiguous slices with a slice thickness of 3.0 mm without an interslice gap; isotropic voxel size of 3.0 x 3.0 x 3.0 mm on a base matrix of 64 x 64 pixels). The functional scan comprised one run of 150 trials (75 cognitive trials and 75 physical trials, randomly intermixed). A total of 585 volumes were collected, with the first 6 volumes removed to allow for steady-state tissue magnetisation. We also acquired a structural T1 image with an MPRAGE sequence for anatomical localisation and gradient-echo field maps to correct for geometric distortions caused by magnetic field inhomogeneities.

### Analyses

#### Physical performance in the cognitive effort task

Previous studies have manipulated physical effort by increasing the number of button presses required across different effort levels. In order to ensure that the perception of higher effort in our cognitive task could not be accounted for by higher physical effort in the form of a greater number of button presses, we performed a repeated-measures ANOVA on the number of button presses across each cognitive effort level. The mean number of button presses across the six effort levels was 2.84 ± 0.05 (range 2.7 to 2.9). The ANOVA showed a significant main effect (*F*(5,165) = 3.65, *p* < .005), but the only significant difference was between effort levels 2 and 6, which was in the opposite direction, with the lower effort level being associated with slightly more button presses than the higher (2.93 ± 0.04 versus 2.74 ± 0.07, *p* < .05). Together, this shows that the number of button presses varied within a very narrow range across effort levels and that increasing physical effort could not account for the subsequent effort discounting effects we observed.

#### Subjective effort—NASA task load index

To confirm that participants’ perception of effort increased with each increment of effort level in the corresponding domain, participants completed the NASA Task Load Index—a questionnaire used to assess subjective perceptions of task demand [36]. Specifically, we examined the difference between the mental and physical demand perceived for each effort level for the cognitive and physical tasks separately (S6 Fig). A repeated-measures ANOVA on these data revealed that each task was considered significantly more demanding in the corresponding domain (*F*(1, 33) = 131.9, *p* < .001) and that the perceived demand in the corresponding domain increased as a function of effort (*F*(2.2, 72.8) = 45.1, *p* < .001). These data confirm that, subjectively, participants perceived the physical effort task to be more physically than mentally demanding and the cognitive effort task to be more mentally than physically demanding.

#### Computational modelling of choice behaviour

The shape of a discounting function reflects how the perception of increasing effort affects choice behaviour. For example, linear models predict constant discounting as effort increases. In contrast, a hyperbolic or exponential model predicts that changes at the lower levels of effort will have greater impact than changes at higher levels, whereas a parabolic model would predict the opposite. These functions have been used in several previous studies to fit effort discounting in the physical domain [5, 14, 33]. In particular, perceived physical effort in constant-force tasks with a hand dynamometer has been shown to increase parabolically as a function of the target force (Stevens’ power law [33, 80]). In the cognitive effort literature, effort discounting has been less thoroughly investigated.

As detailed in the main text, we fitted linear, hyperbolic, parabolic, and exponential functions to participants’ choices in the cognitive and physical effort tasks using a *softmax* function and maximum likelihood estimation and determined the best-fitting model with an AIC and BIC.

Linear:                 *SV*(*t*) = *R*(*t*)∙(1−*k*∙*E*(*t*))
Hyperbolic:         $SV(t)=R(t)∙\frac{1}{1+k∙E(t)}$
Parabolic:             *SV*(*t*) = *R*(*t*)−*k*∙*E*(*t*)^2^
Exponential:         *SV*(*t*) = *R*(*t*)∙*e*^−*k*∙*E*(*t*)^

In these models, S*V*(*t*) represents the subjective value of the offer on trial *t*, *R* is the reward in credits (2, 4, 6, 8, or 10), *E* is the effort level (0.2, 0.4, 0.6, 0.8, or 1.0), and *k* is a subject-specific discounting parameter, which describes the steepness of each individual’s discounting function. Thus, the higher the *k* value, the steeper the discount function. Note that each individual’s discounting function is referenced to the SV of the baseline offer (one). This modelling approach allows us to determine whether individuals were equally willing to exert different types of effort to obtain the identical level of reward, with rewards matched across the cognitive and physical tasks.

A total of 36 models were fitted using a *softmax* function and maximum likelihood estimation (Fig 3). Note that this represents a comparison of many more models than most other carefully conducted computational studies of effort-based decisions or reward-based decision making in general (e.g., [5, 33]). Importantly, we did not compare models in which we placed a weight on individuals’ sensitivity to rewards - as rewards were perfectly matched across the two tasks, any differences in the willingness to exert effort between the two domains must have been due to difference in how the effort was devaluing rewards and not changes in reward sensitivity.

The *softmax* function was defined as:

$$\mathrm{Pr}\left(i\right)=\frac{e^{\beta ∙{SV}_{i}}}{{e^{\beta }+e}^{\beta ∙{SV}_{i}}}$$

in which Pr(*i*) represents the probability of choosing option *i* that has a subjective value of *SV*, and *β* is the *softmax* parameter that defines the stochasticity of each participant’s choices. Four models assumed that cognitive and physical effort discounting are both described by identical functions with identical discounting parameters and *softmax β*s. A further 16 modelled effort discounting with separate discounting parameters but a single *softmax β* for both domains. The final 16 modelled separate discounting parameters and separate *softmax β*s for each domain—these therefore assumed that rewards were distinctly devalued by both cognitive and physical effort and that choices were also differentially stable within each domain.

We used the *fminsearch* function in Matlab to fit each model 20 times with new random starting values for each parameter, with estimations always converging. Model fits were compared using the BIC and the AIC [34], which penalise models for the number of free parameters that are estimated to ensure that models with more parameters are not overfitted. Although it is sometimes difficult to interpret small differences in AIC or BIC values in a single model comparison, both analyses revealed the identical winning model (Fig 3, S5 Fig). A nonparametric (Spearman’s) correlation analysis was performed between the *k* values for cognitive and physical effort but was not significant (*ρ* = 0.22, *p* = .20).

#### fMRI analyses for cognitive and physical effort valuation

Data were processed and analysed using SPM8 (Wellcome Department of Imaging Neuroscience, Institute of Neurology, London, United Kingdom; http://www.fil.ion.ucl.ac.uk/spm), implemented in Matlab (Mathworks Inc., US). The EPI images were first realigned and coregistered to each participant’s own anatomical image using a least-squares approach and six-parameter, rigid-body spatial transformations. The structural image was processed using a unified segmentation procedure combining segmentation, bias correction, and spatial normalisation to the standard MNI template. The same normalisation parameters were then used to normalise the EPI images, which were then spatially smoothed using an isotropic Gaussian kernel at 8 mm full-width at half-maximum.

Each participant’s data was modelled using fixed-effects analyses in SPM8. The effects of the experimental paradigm were estimated for each participant on a voxel-by-voxel basis using the principles of the general linear model (GLM). Predictor functions were formed by modelling the onsets of the events of interest with a stick (delta) function convolved with the canonical haemodynamic response function. Low-frequency noise was removed with a 128 s high-pass filter. The GLM included two regressors of interest: one modelled the onset of the offer cue, and the other modelled the onset of the response cue.

To examine whether activity in any voxel covaried parametrically with the valuation of participants’ chosen option, we created parametric modulators for the offer cue event-related regressor. We used the difference in value between the SV of the chosen offer and the value of the baseline. Although this value difference closely corresponds to the value of the variable option, many previous studies have suggested that many regions signal value relative to the alternative rather than simply the value of the chosen offer [37, 51]. We therefore used these value differences as determined by the modelling analyses above and scaled the amplitude of the HRF to correspond with the SV difference. In separate GLMs, we also created parametric modulators using the raw effort and reward magnitudes of each trial offer (S8 Fig). We modelled trials with an omitted (missed) response as separate regressors, which were not analysed. The six head motion parameters derived during realignment (three translations and three rotations) were incorporated as additional confounding regressors. Regression coefficients were estimated at the subject level using the standard restricted minimum-likelihood estimation implemented in SPM8.

To identify areas that were only active for cognitive and physical effort valuations, SPM contrast images from the first level were input into a second-level *t*-test for each domain separately. *F*-contrasts were then applied at the second level to identify areas that varied with the parametric modulators for cognitive and physical effort separately, either positively or negatively. We considered significant those voxels that survived correction at a family-wise error rate of *p* < .05. In order to identify areas that were active for the conjunction of cognitive and physical effort SVs, first-level SPM contrast images with SV as a parametric modulator were input into a second-level, full-factorial random effects ANOVA with pooled variance, with domain (cognitive versus physical) as a within-subjects variable. As described in the main text, *F*-contrasts were applied at the second level to look for areas in which activity varied statistically, with a linear combination of the betas corresponding to the parametric modulators across the blocks. These *F*-contrasts were then performed as a conjunction analysis at the level of the whole brain. Parameter estimates for the preceding analyses are plotted in Fig 4 for clusters that were significant at *p*_FWE_ < .05.

We then determined whether the amygdala was differentially sensitive to cognitive versus physical effort by performing *t*-tests on SV between the cognitive and physical tasks on an amygdala SVC region. Because previous studies have emphasised the importance of the VS and vmPFC in processing value, we also probed these areas using regions of interest based on coordinates from previous studies [20, 39]. Specifically, we generated spheres of 8 mm diameter around the peak coordinate of voxels found to encode decision values in the paracingulate gyrus (–2, 40, –6), subcallosal cortex (4, 30, –16), frontopolar cortex (–4, 60, –10), dorsal posterior cingulate cortex (0, –34, 42), left orbitofrontal cortex (–24, 32, –16), and nucleus accumbens (–10, 8, –6; 10, 12, –10; also –10, 4, –2 [20]). The only results to emerge from these analyses were weak clusters of activity for the cognitive task alone within the left VS (–12, 2, 0, *k* = 6, *Z* = 4.21, *p*_unc_ < .001) and subcallosal cortex (6, 28, –14, *k* = 4, *Z* = 3.89, *p*_unc_ < .001). No statistically significant clusters were found for the conjunction of cognitive and physical effort, nor for the comparisons of cognitive > physical or physical > cognitive.

#### Control fMRI analyses

In a supplementary analysis, we performed a similar analysis using subjective value as a parametric modulator but now time-locked to the onset of the choice cue (rather than the offer cue). This analysis revealed very little activity for either the cognitive or physical effort conditions, with only motor (cerebellar) regions being significant in the conjunction analysis (S1 Table).

To ensure that perceived risk could not account for our results, we used three different measures of risk as parametric modulators of participants’ choices at the onset of the offer cue: (1) the proportion of errors (i.e., unrewarded trials) at each effort level for each domain in the training session, (2) *d’* for each effort level as a composite measure of performance in the cognitive effort task (*Z*(Hit)—*Z*(False Alarm)), and (3) the total time spent within the required force window at each effort level as an index of performance in the physical effort task. These analyses did not reveal any significant voxels in the domain-independent regions signalling SV or the amygdala, even at uncorrected thresholds of *p* < .001.

Next, we performed three separate analyses to exclude the possibility that choice difficulty could explain our results:

1. Given that decision reaction time covaries with decision difficulty (with more difficult decisions taking longer), we performed a second analysis using decision reaction time (from the onset of the choice cue) as a parametric modulator time-locked to the onset of the offer cue.
2. In addition, we used the probability, *p*, of choice predictions estimated from our *softmax* model to calculate decision difficulty. This probability takes into account individuals’ *softmax* parameters, which we can then use to estimate difficulty as -|*p*-0.5|. More difficult decisions should be reflected as probabilities closer to 0.5 (or difficulty = 0), while easier decisions should be reflected as probabilities closer to 0 or 1 (difficulty < 0). Analogous to the primary analyses, we entered the difficulty level on each trial as parametric modulators time-locked to the onset of the offer cue. *F*-contrasts were then applied at the second level to identify areas in which activity varied statistically with this regressor.

Crucially, for all of these measures of difficulty, there were no significant clusters at the identical threshold used in the principal analyses on subjective value (*p*_FWE_ < .05) or even at more liberal uncorrected thresholds of *p*_unc_ < .001. Furthermore, no significant clusters were associated with cognitive choice difficulty, either in the right amygdala or in the remainder of the brain, suggesting that the amygdala activity was uniquely related to the subjective valuation of cognitive effort and not driven by risk or decision difficulty.

Finally, as discussed in the main manuscript, the finding of domain-general areas does not hinge on the nature of the specific functions that comprised the winning model. Nevertheless, an interesting question relating to generalisability is whether similar results would be obtained if only a single function were used to model cognitive and physical effort. We therefore ran additional exploratory analyses using models that would offer the best fits if only a single function could be used across both domains (i.e., the lowest AIC or BIC along the antidiagonal of the middle and right matrices of Fig 3 and S5 Fig). Given such constraints, an exponential function provided the best model fits (but we note that these fits were inferior to that of the winning model described in the paper).

Using procedures analogous to those in the primary analyses, we computed SVs using an exponential function, with separate analyses conducted for models using single and separate *softmax β*s. In examining the conjunction of areas in which activity varied with cognitive and physical SVs for exponential functions, these analyses revealed very similar overlapping areas of activation to the main analyses, specifically in the bilateral parietal cortex, dorsal ACC, bilateral insula, and right dlPFC. These areas were identified at slightly lower levels of significance compared to our main analyses (*p* = .001, uncorrected), which is to be expected given that these were less superior models. Nevertheless, these additional analyses are consistent overall with the finding that the valuation of cognitive and physical effort rely on largely overlapping neural substrates.

Turning to the amygdala result, a related question is whether the amygdala preference for cognitive over physical effort valuation was driven by the different underlying functions. To confirm that this was not the case, we repeated our analyses using an amygdala SVC on the exponential/exponential models described above. This again resulted in significant amygdala activation, with the identical peak voxel of activation as in the main analysis (22, –12, –10) at family-wise corrected error rates (*p*_FWE_ < .05). These additional analyses demonstrate that the primary amygdala results could not simply be due to differing functions describing cognitive and physical effort discounting. However, given that these analyses are exploratory and are based on models that may not have been the most appropriate fits to our task, future studies should aim to replicate our findings using tasks that do result in similar discounting functions across separate domains.

## Supporting information

S1 Fig Success rates for the cognitive and physical effort tasks in the prescan training session. Participants were rewarded on over 80% of trials in both tasks. Underlying data can be found in S1 Data.
(TIF)

S2 Fig Median reaction times for decisions made in the scanner, as a function of (A) effort and (B) reward. There were no significant conditional differences. Underlying data for panels A–B can be found in S1 Data.
(TIF)

S3 Fig Logistic regression on choice behaviour for the (A) cognitive and (B) physical effort tasks. Effort, reward, and reinforcement rates were entered as covariates in a separate general linear model for each participant. Normalised *t*-statistics (*β*/SE(*β*)) showed that effort significantly predicted choice behaviour in the negative direction and reward in the positive direction for both the cognitive and physical tasks. Importantly, reinforcement rates did not significantly predict choice behaviour, allowing us to exclude probability discounting as an explanation for participants’ decisions. Underlying data for panels A–B can be found in S1 Data.
(TIF)

S4 Fig An example of two participants who differentially valued cognitive and physical effort. (A) Subject 1 was significantly more motivated physically than cognitively, in contrast to (B) Subject 2, who showed the reverse pattern. Such individual differences are not observed when data are collapsed across the group. Underlying data for panels A–B can be found in S1 Data.
(TIF)

S5 Fig Results of model comparisons with a Bayesian Information Criterion, revealing the identical pattern of results and identical winning model compared to when using an Akaike Information Criterion. Underlying data can be found in S1 Data.
(TIF)

S6 Fig Perceived demand in each of the two tasks, as assessed with the NASA Task Load Index. Participants reported the subjective perceived demand for each level of each task to be higher in the corresponding domain. Underlying data can be found in S1 Data.
(TIF)

S7 Fig Whole-brain analysis showing areas in which activity parametrically varied with SV in the cognitive (blue) and physical (red) tasks (violet indicates areas of overlap). (A) Whole-brain render, showing foci in the right intraparietal sulcus (IPS) and the dorsolateral prefrontal cortex (dlPFC), including the middle frontal gyrus (MFG), adjacent inferior frontal sulcus (IFS), and superior frontal gyrus (SFG). (B) Coronal section showing activity in the anterior cingulate cortex (ACC) and adjacent dorsomedial prefrontal cortex (dmPFC), the IFS, and the right insula. (C) Sagittal sections showing ACC/dmPFC, IPS, dlPFC and insula activity.
(TIF)

S8 Fig Activity in most of the domain-general SV regions varied positively with the effort level of the offer and negatively with reward level. Parameter estimates are shown for each domain-general area as a function of the following: the SV difference between the chosen option and the baseline, the effort level of the offer, and the reward level of the offer. Data are plotted separately for (A) cognitive effort and (B) physical effort. Underlying data for panels A–B can be found in S1 Data.
(TIF)

S9 Fig Results of control fMRI analyses for choice difficulty, using (A) choice reaction time and (B) choice probabilities from the *softmax* model. Each of these measures were entered into a general linear model as parametric regressors time-locked to the onset of the offer cue. None of the areas that signalled subjective value signalled choice difficulty. Underlying data for panels A–B can be found in S1 Data.
(TIF)

S10 Fig Results of control fMRI analyses for risk, using (A) error rates and (B) performance measures for each effort level in each task during the prescan training session. *d’* was used as a composite measure of performance in the cognitive effort task, and the time spent within the required force window was used as an index of performance for the physical effort task. Each measure was entered into a general linear model as parametric regressors time-locked to the onset of the offer cue. None of the areas that signalled subjective value signalled risk. Underlying data for panels A–B can be found in S1 Data.
(TIF)

S1 Table Areas sensitive to the SV difference between the chosen option and baseline, time-locked to the onset of the response prompt. Data are shown for (A) cognitive effort, (B) physical effort, and (C) the conjunction of both domains. Clusters are significant at a voxel-wise threshold of *p* < .05, corrected for family-wise error. Coordinates are given in MNI space.
(DOCX)

S1 Data Provides underlying data for Figs 2, 3, 4D and 5 and S1–S6 and S8–S10 Figs. (DOCX)

## Abbreviations

ACC: anterior cingulate cortex
AI: anterior insula
AIC: Akaike Information Criterion
BA: Brodmann area
BIC: Bayesian Information Criterion
BOLD: blood oxygen level–dependent
dACC: dorsal anterior cingulate cortex
dlPFC: dorsolateral prefrontal cortex
dmPFC: dorsomedial prefrontal cortex
fMRI: functional magnetic resonance imaging
FWE: family-wise error
IPS: intraparietal sulcus
MD: multiple demand
MNI: Montreal Neurological Institute
MVC: maximum voluntary contraction
ROI: region of interest
RSVP: rapid serial visual presentation
SV: subjective value
vmPFC: ventromedial prefrontal cortex
VS: ventral striatum

## References

[1] Robbins T, Everitt B. Neurobehavioural mechanisms of reward and motivation. Current Opinion in Neurobiology. 1996;6:228–36. 8725965 10.1016/s0959-4388(96)80077-8

[2] Salamone JD, Correa M. The mysterious motivational functions of mesolimbic dopamine. Neuron. 2012;76(3):470–85. 10.1016/j.neuron.2012.10.021 23141060 PMC4450094

[3] Varazzani C, San-Galli A, Gilardeau S, Bouret S. Noradrenaline and dopamine neurons in the reward/effort trade-off: A direct electrophysiological comparison in behaving monkeys. Journal of Neuroscience. 2015;35(20):7866–77. 10.1523/JNEUROSCI.0454-15.2015 25995472 PMC6795183

[4] Apps M, Grima L, Manohar S, Husain M. The role of cognitive effort in subjective reward devaluation and risky decision-making. Scientific Reports. 2015;5:16880 10.1038/srep16880 26586084 PMC4653618

[5] Klein-Flügge M, Kennerley S, Saraiva A, Penny W, Bestmann S. Behavioral modeling of human choices reveals dissociable effects of physical effort and temporal delay on reward devaluation. PLoS Comput Biol. 2015;11(3):e1004116 10.1371/journal.pcbi.1004116 25816114 PMC4376637

[6] Westbrook A, Braver T. Cognitive effort: A neuroeconomic approach. Cognitive, Affective, and Behavioral Neuroscience. 2015;15(2):395–415. 10.3758/s13415-015-0334-y PMC4445645 25673005

[7] Manohar SG, Chong TT-J, Apps MA, Batla A, Stamelou M, Jarman PR, et al Reward pays the cost of noise reduction in motor and cognitive control. Current Biology. 2015;25(13):1707–16. 10.1016/j.cub.2015.05.038 26096975 PMC4557747

[8] Chong TT-J, Bonnelle V, Husain M. Quantifying motivation with effort-based decision-making paradigms in health and disease. Progress in Brain Research. 2016;229:71–100. 10.1016/bs.pbr.2016.05.002 27926453

[9] Levy D, Glimcher P. The root of all value: a neural common currency for choice. Current Opinion in Neurobiology. 2012;22:1027–38. 10.1016/j.conb.2012.06.001 22766486 PMC4093837

[10] Peters J, Büchel C. Neural representations of subjective reward value. Behavioural Brain Research. 2010;213:135–41. 10.1016/j.bbr.2010.04.031 20420859

[11] Croxson P, Walton M, O'Reilly J, Behrens T, Rushworth M. Effort-based cost-benefit valuation and the human brain. Journal of Neuroscience. 2009;29(14):4531–41. 10.1523/JNEUROSCI.4515-08.2009 19357278 PMC2954048

[12] Hosking J, Floresco S, Winstanley C. Dopamine antagonism decreases willingness to expend physical, but not cognitive, effort: A comparison of two rodent cost/benefit decision-making tasks. Neuropsychopharmacology. 2015;40:1005–15. 10.1038/npp.2014.285 25328051 PMC4330516

[13] Kurniawan I, Seymour B, Talmi D, Yoshida W, Chater N, Dolan R. Choosing to make an effort: the role of striatum in signaling physical effort of a chosen action. Journal of Neurophysiology. 2010;104(1):313–21. 10.1152/jn.00027.2010 20463204 PMC2904211

[14] Prévost C, Pessiglione M, Météreau E, Cléry-Melin M-L, Dreher J-C. Separate valuation subsystems for delay and effort decision costs. Journal of Neuroscience. 2010;30:14080–90. 10.1523/JNEUROSCI.2752-10.2010 20962229 PMC6634773

[15] Skvortsova V, Palminteri S, Pessiglione M. Learning to minimize efforts versus maximizing rewards: computational principles and neural correlates. Journal of Neuroscience. 2014;34(47):15621–30. 10.1523/JNEUROSCI.1350-14.2014 25411490 PMC6608437

[16] Walton M, Bannerman D, Rushworth M. The role of rat medial frontal cortex in effort-based decision making. Journal of Neuroscience. 2002;22:10996–1003. 12486195 10.1523/JNEUROSCI.22-24-10996.2002 PMC6758435

[17] Apps M, Ramnani N. The anterior cingulate gyrus signals the net value of others' rewards. Journal of Neuroscience. 2014;34(18):6190–200. 10.1523/JNEUROSCI.2701-13.2014 24790190 PMC4004808

[18] Bonnelle V, Manohar S, Behrens T, Husain M. Individual differences in premotor brain systems underlie behavioral apathy. Cerebral Cortex. 2016;26:807–19. 10.1093/cercor/bhv247 26564255 PMC4712805

[19] Chong TT-J, Bonnelle V, Manohar S, Veromann K-R, Muhammed K, Tofaris G, et al Dopamine enhances willingness to exert effort for reward in Parkinson's disease. Cortex. 2015;69:40–6. 10.1016/j.cortex.2015.04.003 25967086 PMC4533227

[20] Schmidt L, Lebreton M, Cléry-Melin M-L, Daunizeau J, Pessiglione M. Neural mechanisms underlying motivation of mental versus physical effort. PLoS Biol. 2012;10(2):e1001266 10.1371/journal.pbio.1001266 22363208 PMC3283550

[21] Hartmann MN, Hager OM, Reimann AV, Chumbley JR, Kirschner M, Seifritz E, et al Apathy but not diminished expression in schizophrenia is associated with discounting of monetary rewards by physical effort. Schizophrenia Bulletin. 2015;41(2):503–12. 10.1093/schbul/sbu102 25053653 PMC4332944

[22] Kolling N, Rushworth M. What's worth the risk? A neural circuit for trade-offs. Cell. 2015;161(6):1243–4. 10.1016/j.cell.2015.05.031 26046432

[23] Schonberg T, Bakkour A, Hover A, Mumford J, Nagar L, Perez J, et al Changing value through cued approach: an automatic mechanism of behavior change. Nature Neuroscience. 2014;17:625–30. 10.1038/nn.3673 24609465 PMC4041518

[24] Hunt L, Kolling N, Soltani A, Woolrich M, Rushworth M, Behrens T. Mechanisms underlying cortical activity during value-guided choice. Nature Neuroscience. 2012;15:470–6. 10.1038/nn.3017 22231429 PMC3378494

[25] Floresco S, Ghods-Sharifi S. Amygdala-prefrontal cortical circuitry regulates effort based decision making. Cerebral Cortex. 2007;17:251–60. 10.1093/cercor/bhj143 16495432

[26] Ghods-Sharifi S, St Onge J, Floresco S. Fundamental contribution by the basolateral amygdala to different forms of decision making. Journal of Neuroscience. 2009;29(16):5251–9. 10.1523/JNEUROSCI.0315-09.2009 19386921 PMC6665478

[27] Hosking J, Cocker P, Winstanley C. Dissociable contributions of anterior cingulate cortex and basolateral amygdala on a rodent cost/benefit decision-making task of cognitive effort. Neuropsychopharmacology. 2014;39:1558–67. 10.1038/npp.2014.27 24496320 PMC4023153

[28] Schultz W. Updating dopamine reward signals. Current Opinion in Neurobiology. 2013;23(2):229–38. 10.1016/j.conb.2012.11.012 23267662 PMC3866681

[29] Yantis S, Schwarzbach J, Serences J, Carlson R, Steinmetz M, Pekar J, et al Transient neural activity in human parietal cortex during spatial attention shifts. Nature Neuroscience. 2002;5:995–1002. 10.1038/nn921 12219097

[30] Kable J, Glimcher P. The neural correlates of subjective value during intertemporal choice. Nature Neuroscience. 2007;10(12):1625–33. 10.1038/nn2007 17982449 PMC2845395

[31] Pine A, Seymour B, Roiser J, Bossaerts P, Friston K, Curran H, et al Encoding of marginal utility across time in the human brain. Journal of Neuroscience. 2009;29(30):9575–81. 10.1523/JNEUROSCI.1126-09.2009 19641120 PMC2816907

[32] Shenhav A, Straccia M, Cohen J, Botvinick M. Anterior cingulate engagement in a foraging context reflects choice difficulty, not foraging value. Nature Neuroscience. 2014;17(9):1249–54. 10.1038/nn.3771 25064851 PMC4156480

[33] Hartmann M, Hager O, Tobler P, Kaiser S. Parabolic discounting of monetary rewards by physical effort. Behavioral Processes. 2013;100:192–6. 10.1016/j.beproc.2013.09.014 24140077

[34] Akaike H. A new look at the statistical model identification. IEEE Transactions on Automatic Control. 1974;19(6):716–23.

[35] Schwartz GE. Estimating the dimension of a model. Annals of Statistics. 1978;6(2):461–4.

[36] Hart S, Staveland L. Development of NASA-TLX (Task Load Index): Results of empirical and theoretical research. Advances in Psychology. 1988;52:139–83.

[37] Nicolle A, Klein-Flügge M, Hunt L, Vlaev I, Dolan R, Behrens T. An agent independent axis for executed and modeled choice in medial prefrontal cortex. Neuron. 2012;75:1114–21. 10.1016/j.neuron.2012.07.023 22998878 PMC3458212

[38] Nichols T, Brett M, Andersson J, Wager T, Poline J. Valid conjunction inference with the minimum statistic. Neuroimage. 2005;25(653–660). 10.1016/j.neuroimage.2004.12.005 15808966

[39] Clithero J, Rangel A. Informatic parcellation of the network involved in the computation of subjective value. Social Cognitive and Affective Neuroscience. 2014;9:1289–302. 10.1093/scan/nst106 23887811 PMC4158359

[40] Salamone JD, Yohn SE, López-Cruz L, San Miguel N, Correa M. Activational and effort-related aspects of motivation: neural mechanisms and implications for psychopathology. Brain. 2016;139(5):1325–47. 27189581 10.1093/brain/aww050 PMC5839596

[41] Wassum KM, Izquierdo A. The basolateral amygdala in reward learning and addiction. Neuroscience and Biobehavioral Reviews. 2015;57:271–83. 10.1016/j.neubiorev.2015.08.017 26341938 PMC4681295

[42] Ghashghaei HT, Hilgetag CC, Barbas H. Sequence of information processing for emotions based on the anatomic dialogue between prefrontal cortex and amygdala. Neuroimage. 2007;34(3):905–23. 10.1016/j.neuroimage.2006.09.046 17126037 PMC2045074

[43] Vassena E, Silvetti M, Boehler C, Achten E, Fias W, Verguts T. Overlapping neural systems represent cognitive effort and reward anticipation. PLoS ONE. 2014;9(3):e91008 10.1371/journal.pone.0091008 24608867 PMC3946624

[44] Burke C, Brünger C, Kahnt T, Park S, Tobler P. Neural integration of risk and effort costs by the frontal pole: only upon request. Journal of Neuroscience. 2013;33(4):1706–13. 10.1523/JNEUROSCI.3662-12.2013 23345243 PMC6618754

[45] Padoa-Schioppa C, Assad J. Neurons in the orbitofrontal cortex encode economic value. Nature. 2006;441:223–6. 10.1038/nature04676 16633341 PMC2630027

[46] Chong TT-J, Husain M. The role of dopamine in the pathophysiology and treatment of apathy. Progress in Brain Research. 2016;229:389–426. 10.1016/bs.pbr.2016.05.007 27926449

[47] Roesch M, Taylor A, Schoenbaum G. Encoding of time-discounted rewards in orbitofrontal cortex is independent of value. Neuron. 2006;51:509–20. 10.1016/j.neuron.2006.06.027 16908415 PMC2561990

[48] Rudebeck P, Walton M, Smyth A, Bannerman D, Rushworth M. Separate neural pathways process different decision costs. Nature Neuroscience. 2006;9(9):1161–8. 10.1038/nn1756 16921368

[49] Ang YS, Manohar S, Apps MA. Commentary: Noradrenaline and Dopamine Neurons in the Reward/Effort Trade-off: A Direct Electrophysiological Comparison in Behaving Monkeys. Frontiers in Behavioral Neuroscience. 2015;9:310 10.3389/fnbeh.2015.00310 26635560 PMC4644795

[50] Shenhav A, Botvinick M, Cohen J. The expected value of control: an integrative theory of anterior cingulate cortex function. Neuron. 2013;79(2):217–40. 10.1016/j.neuron.2013.07.007 23889930 PMC3767969

[51] Kolling N, Behrens T, Wittmann M, Rushworth M. Multiple signals in anterior cingulate cortex. Current Opinion in Neurobiology. 2016;37:36–43. 10.1016/j.conb.2015.12.007 26774693 PMC4863523

[52] Kolling N, Behrens T, Mars R, Rushworth M. Neural mechanisms of foraging. Science. 2012;336(6077):95–8. 10.1126/science.1216930 22491854 PMC3440844

[53] Hayden B, Pearson J, Platt M. Fictive reward signals in anterior cingulate cortex. Science. 2009;324(5929):948–50. 10.1126/science.1168488 19443783 PMC3096846

[54] Verguts T, Vassena E, Silvetti M. Adaptive effort investment in cognitive and physical tasks: A neurocomputational model. Frontiers in Behavioral Neuroscience. 2015;9:57 10.3389/fnbeh.2015.00057 25805978 PMC4353205

[55] Kennerley S, Dahmubed A, Lara A, Wallis J. Neurons in the frontal lobe encode the value of multiple decision variables. Journal of Cognitive Neuroscience. 2009;21:1162–78. 10.1162/jocn.2009.21100 18752411 PMC2715848

[56] Massar S, Libedinsky C, Weiyan C, Huettel S, Chee M. Separate and overlapping brain areas encode subjective value during delay and effort discounting. Neuroimage. 2015;120:104–13. 10.1016/j.neuroimage.2015.06.080 26163803

[57] Kobayashi S, Nomoto K, Watanabe M, Hikosaka O, Schultz W, Sakagami M. Influences of rewarding and aversive outcomes on activity in macaque lateral prefrontal cortex. Neuron. 2006;51:861–70. 10.1016/j.neuron.2006.08.031 16982429

[58] Leon M, Shadlen M. Effect of expected reward magnitude on the response of neurons in the dorsolateral prefrontal cortex of the macaque. Neuron. 1999;24:415–25. 10571234 10.1016/s0896-6273(00)80854-5

[59] Wallis J, Miller E. Neuronal activity in primate dorsolateral and orbital prefrontal cortex during performance of a reward preference task. European Journal of Neuroscience. 2003;18:2069–81. 14622240 10.1046/j.1460-9568.2003.02922.x

[60] Sokol-Hessner P, Hutcherson C, Hare T, Rangel A. Decision value computation in dlPFC and vmPFC adjusts to the available decision time. European Journal of Neuroscience. 2012;35:1065–74. 10.1111/j.1460-9568.2012.08076.x 22487036 PMC3325500

[61] Platt M, Glimcher P. Neural correlates of decision variables in parietal cortex. Nature. 1999;400:233–8. 10.1038/22268 10421364

[62] Louie K, Glimcher P. Separating value from choice: delay discounting activity in the lateral intraparietal area. Journal of Neuroscience. 2010;30:5498–507. 10.1523/JNEUROSCI.5742-09.2010 20410103 PMC2898568

[63] Sugrue L, Corrado G, Newsome W. Choosing the greater of two goods: Neural currencies for valuation and decision making. Nature Reviews Neuroscience. 2005;6:363–75. 10.1038/nrn1666 15832198

[64] Meyniel F, Safra L, Pessiglione M. How the brain decides when to work and when to rest: Dissociation of implicit-reactive from explicit-predictive computational processes. PLoS Comput Biol. 2014;10:e1003584 10.1371/journal.pcbi.1003584 24743711 PMC3990494

[65] Treadway M, Buckholtz J, Cowan R, Woodward N, Li R, Ansari M, et al Dopaminergic mechanisms of individual differences in human effort-based decision-making. Journal of Neuroscience. 2012;32(18):6170–6. 10.1523/JNEUROSCI.6459-11.2012 22553023 PMC3391699

[66] Borra E, Belmalih A, Calzavara R, Gerbella M, Murata A, Rozzi S, et al Cortical connections of the macaque anterior intraparietal (AIP) area. Cerebral Cortex. 2008;18(5):1094–111. 10.1093/cercor/bhm146 17720686

[67] Mesulam M, Mufson EJ. Insula of the old world monkey. III: Efferent cortical output and comments on function. Journal of Comparative Neurology. 1982;212(1):38–52. 10.1002/cne.902120104 7174907

[68] Petrides M, Pandya DN. Dorsolateral prefrontal cortex: comparative cytoarchitectonic analysis in the human and the macaque brain and corticocortical connection patterns. European Journal of Neuroscience. 1999;11(3):1011–36. 10103094 10.1046/j.1460-9568.1999.00518.x

[69] Vogt BA, Nimchinsky EA, Vogt LJ, Hof PR. Human cingulate cortex: surface features, flat maps, and cytoarchitecture. Journal of Comparative Neurology. 1995;359(3):490–506. 10.1002/cne.903590310 7499543

[70] Duncan J. The multiple-demand (MD) system of the primate brain: mental programs for intelligent behaviour. Trends in Cognitive Sciences. 2010;14(4):172–9. 10.1016/j.tics.2010.01.004 20171926

[71] Fedorenko E, Duncan J, Kanwisher N. Broad domain generality in focal regions of frontal and parietal cortex. Proceedings of the National Academy of Sciences of the United States of America. 2013;110(41):16616–21. 10.1073/pnas.1315235110 24062451 PMC3799302

[72] Holroyd C, Yeung N. Motivation of extended behaviors by anterior cingulate cortex. Trends in Cognitive Science. 2012;16(2):122–8. 10.1016/j.tics.2011.12.008 22226543

[73] Grabenhorst F, Hernádi I, Schultz W. Prediction of economic choice by primate amygdala neurons. Proceedings of the National Academy of Sciences of the United States of America. 2012;109(46):18950–5. 10.1073/pnas.1212706109 23112182 PMC3503170

[74] Waraczynski M. The central extended amygdala network as a proposed circuit underlying reward valuation. Neuroscience and Biobehavioral Reviews. 2006;30(4):472–96. 10.1016/j.neubiorev.2005.09.001 16243397

[75] Jenison R, Rangel A, Oya H, Kawasaki H, Howard M. Value encoding in single neurons in the human amygdala during decision making. Journal of Neuroscience. 2011;31(1):331–8. 10.1523/JNEUROSCI.4461-10.2011 21209219 PMC3028386

[76] Bartra O, McGuire JT, Kable JW. The valuation system: a coordinate-based meta-analysis of BOLD fMRI experiments examining neural correlates of subjective value. NeuroImage. 2013;76:412–27. 10.1016/j.neuroimage.2013.02.063 23507394 PMC3756836

[77] Rahman S, Sahakian B, Cardinal R, Rogers R, Robbins T. Decision making and neuropsychiatry. Trends in Cognitive Sciences. 2001;5:271–7. 11390298 10.1016/s1364-6613(00)01650-8

[78] Chong TT-J. Disrupting the perception of effort with continuous theta burst stimulation. Journal of Neuroscience. 2015;35(39):13269–71. 10.1523/JNEUROSCI.2624-15.2015 26424875 PMC6605477

[79] Deichmann R, Gottfried J, Hutton C, Turner R. Optimized EPI for fMRI studies of the orbitofrontal cortex. NeuroImage. 2003;19(2):430–41. 12814592 10.1016/s1053-8119(03)00073-9

[80] Stevens J, Mack J. Scales of apparent force. Journal of Experimental Psychology. 1959;58(5):405–13. 13834535 10.1037/h0046906

