# Supplementary figures and images for: Neurocomputational mechanisms underlying subjective valuation of effort costs
Source: PLoS Biol. 2017 Feb 24;15(2):e1002598. doi: 10.1371/journal.pbio.1002598 (PMC5325181; doi:10.1371/journal.pbio.1002598)

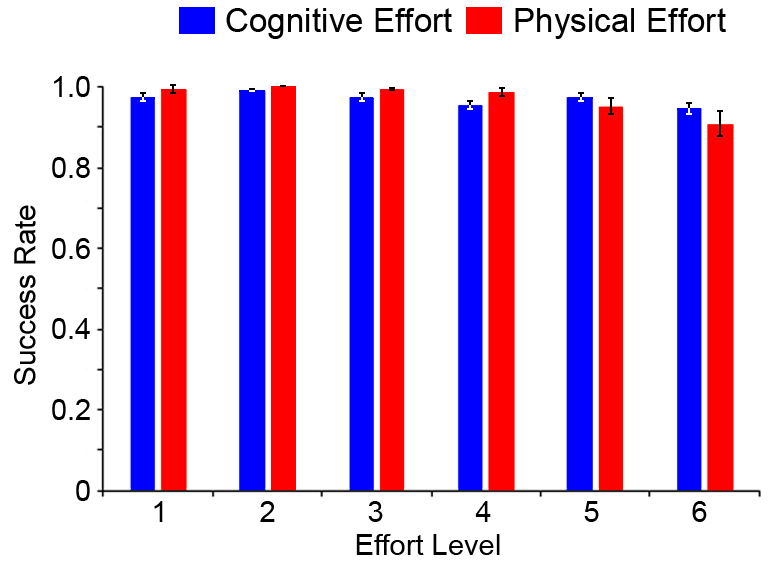

Supplement: S1 Fig — Participants were rewarded on over 80% of trials in both tasks. Underlying data can be found in S1 Data. (TIF) [file pbio.1002598.s001.tif]

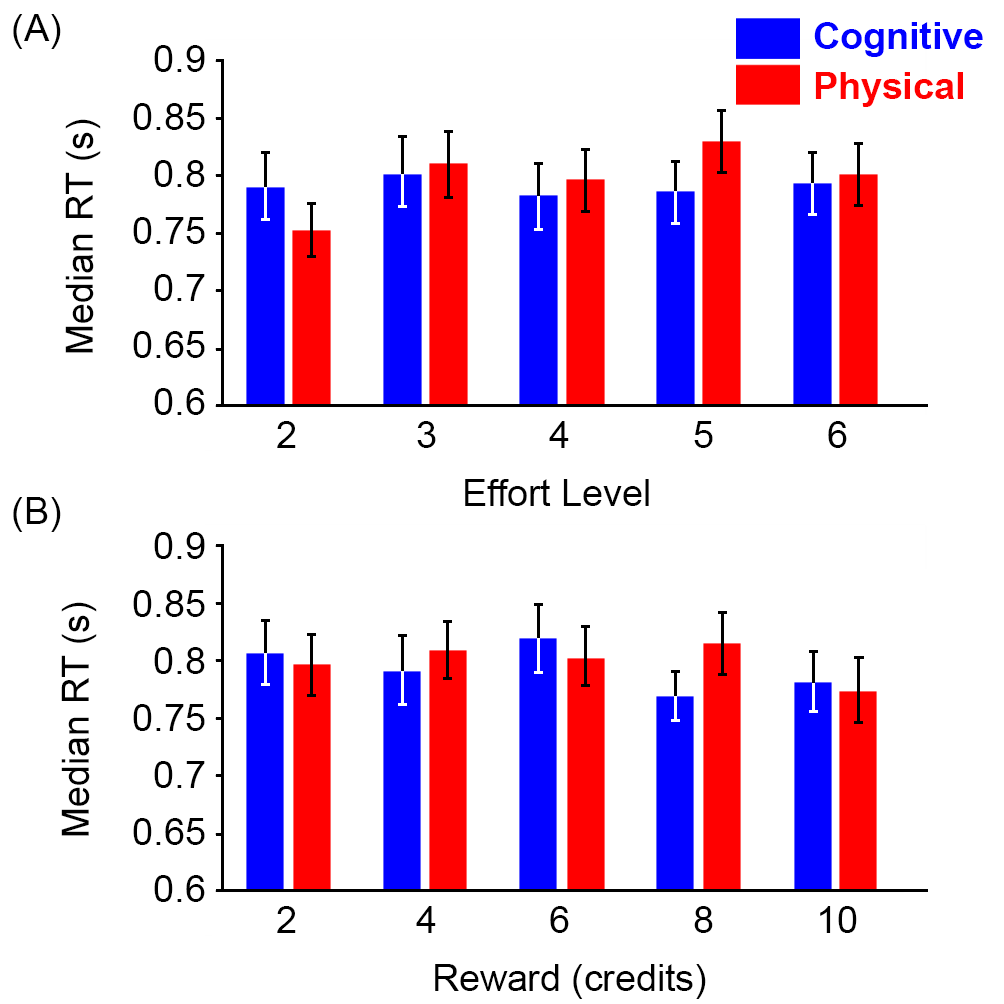

Supplement: S2 Fig — Median reaction times for decisions made in the scanner, as a function of (A) effort and (B) reward. There were no significant conditional differences. Underlying data for panels A–B can be found in S1 Data. (TIF) [file pbio.1002598.s002.tif]

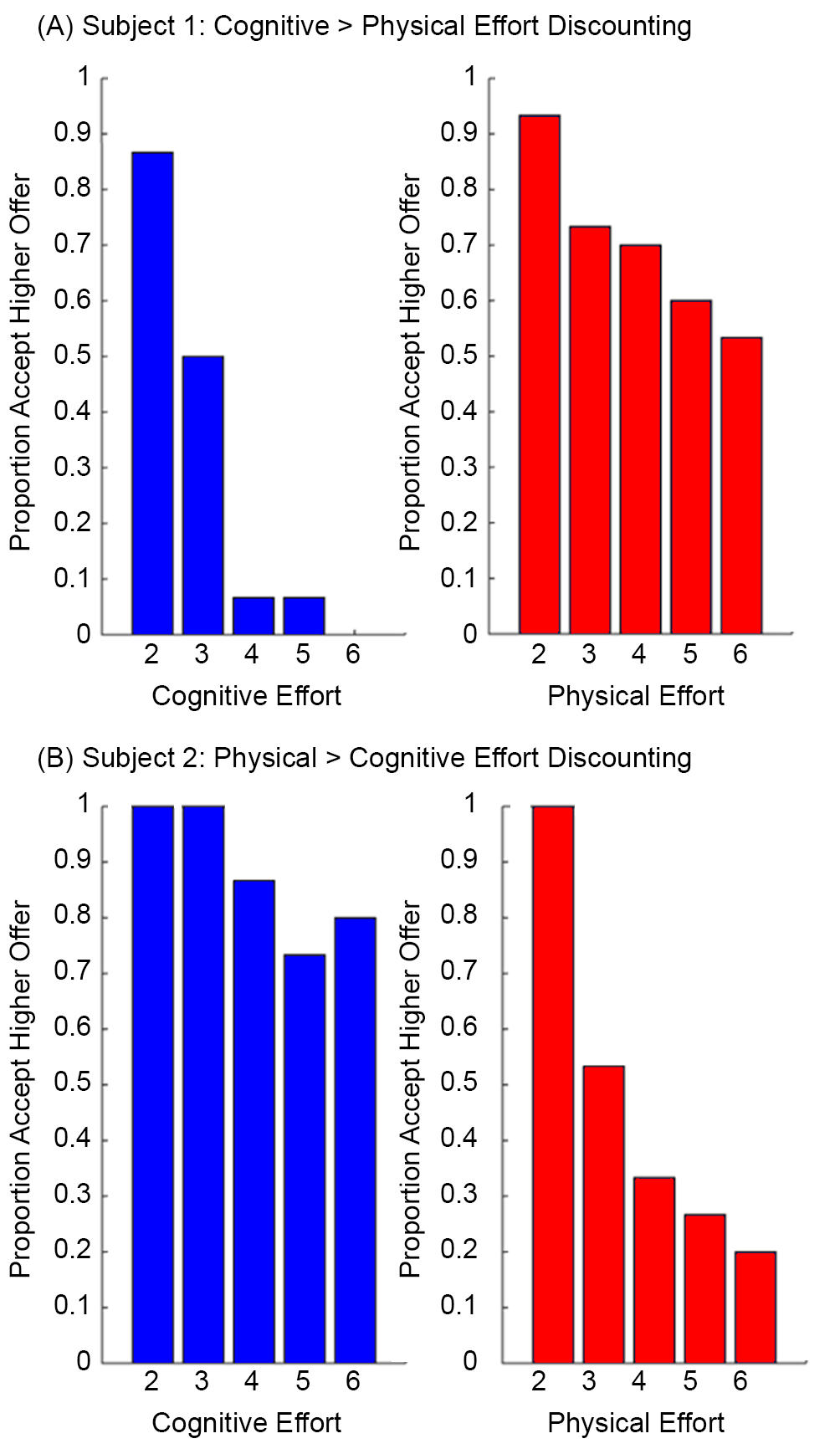

Supplement: S4 Fig — (A) Subject 1 was significantly more motivated physically than cognitively, in contrast to (B) Subject 2, who showed the reverse pattern. Such individual differences are not observed when data are collapsed across the group. Underlying data for panels A–B can be found in S1 Data. (TIF) [file pbio.1002598.s004.tif]

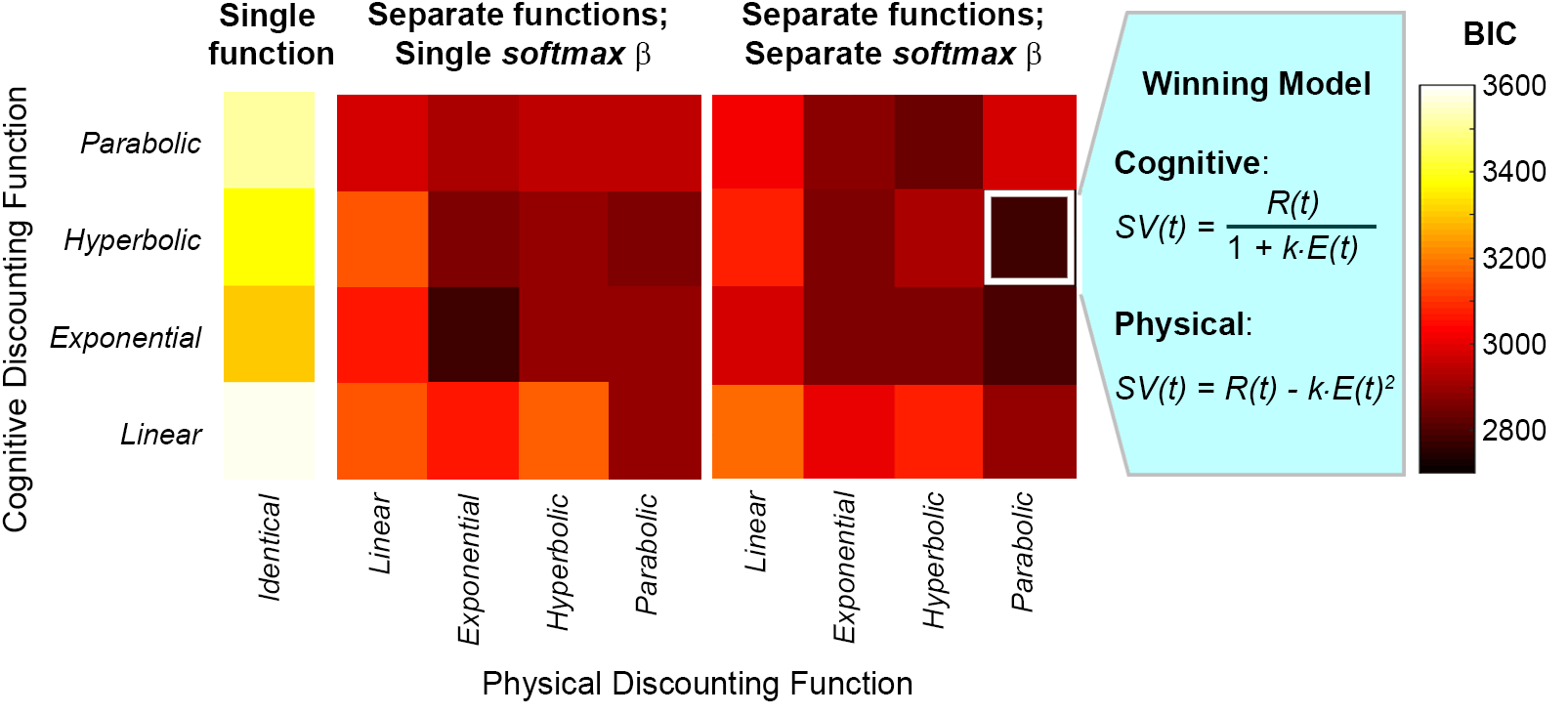

Supplement: S5 Fig — Underlying data can be found in S1 Data. (TIF) [file pbio.1002598.s005.tif]

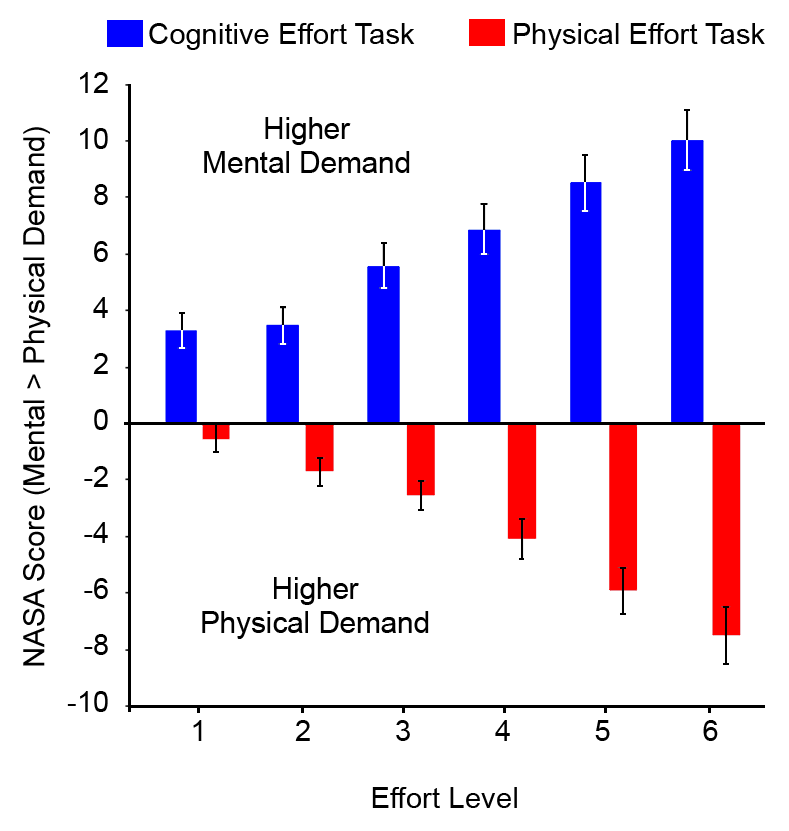

Supplement: S6 Fig — Participants reported the subjective perceived demand for each level of each task to be higher in the corresponding domain. Underlying data can be found in S1 Data. (TIF) [file pbio.1002598.s006.tif]

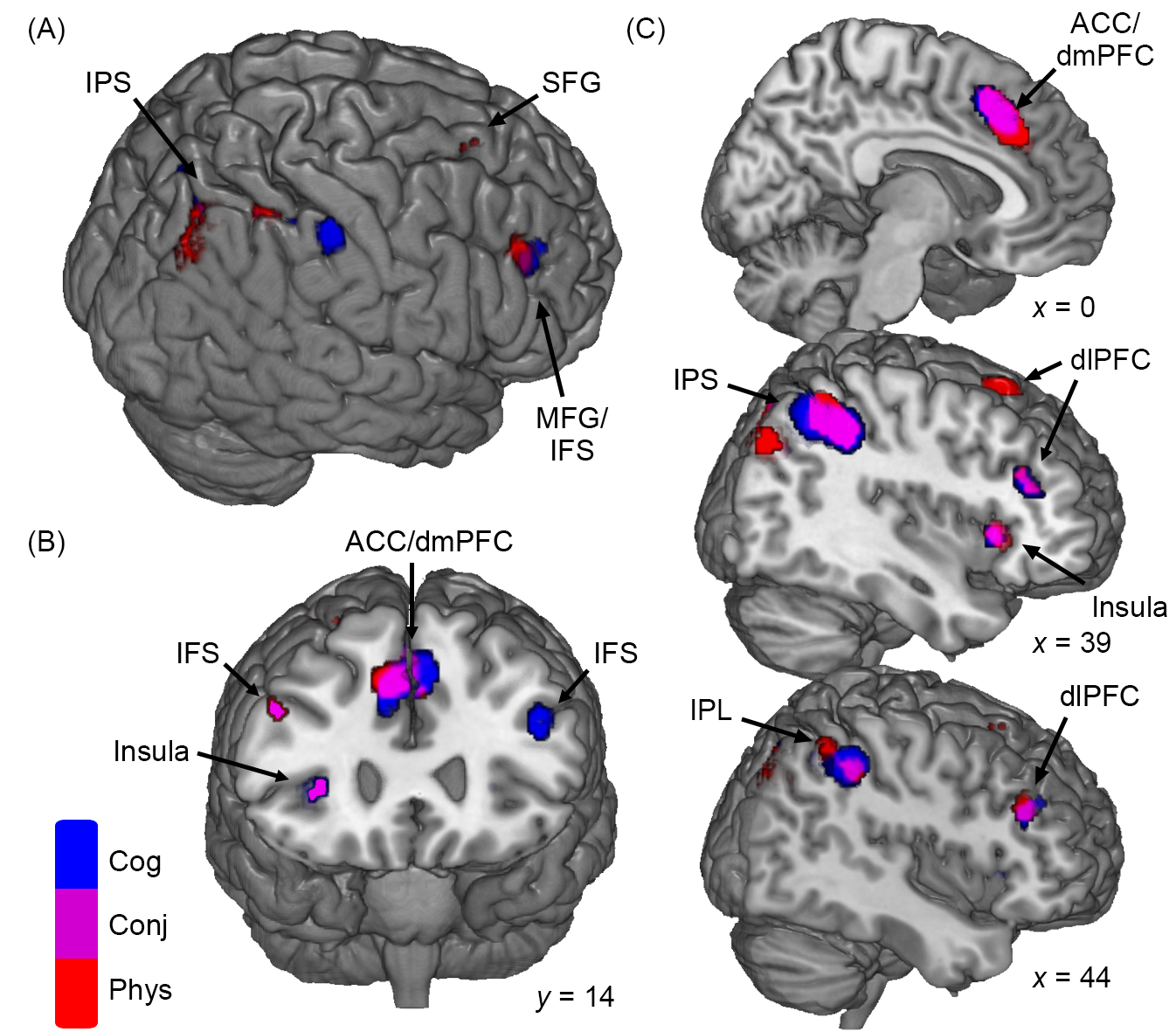

Supplement: S7 Fig — (A) Whole-brain render, showing foci in the right intraparietal sulcus (IPS) and the dorsolateral prefrontal cortex (dlPFC), including the middle frontal gyrus (MFG), adjacent inferior frontal sulcus (IFS), and superior frontal gyrus (SFG). (B) Coronal section showing activity in the anterior cingulate cortex (ACC) and adjacent dorsomedial prefrontal cortex (dmPFC), the IFS, and the right insula. (C) Sagittal sections showing ACC/dmPFC, IPS, dlPFC and insula activity. (TIF) [file pbio.1002598.s007.tif]

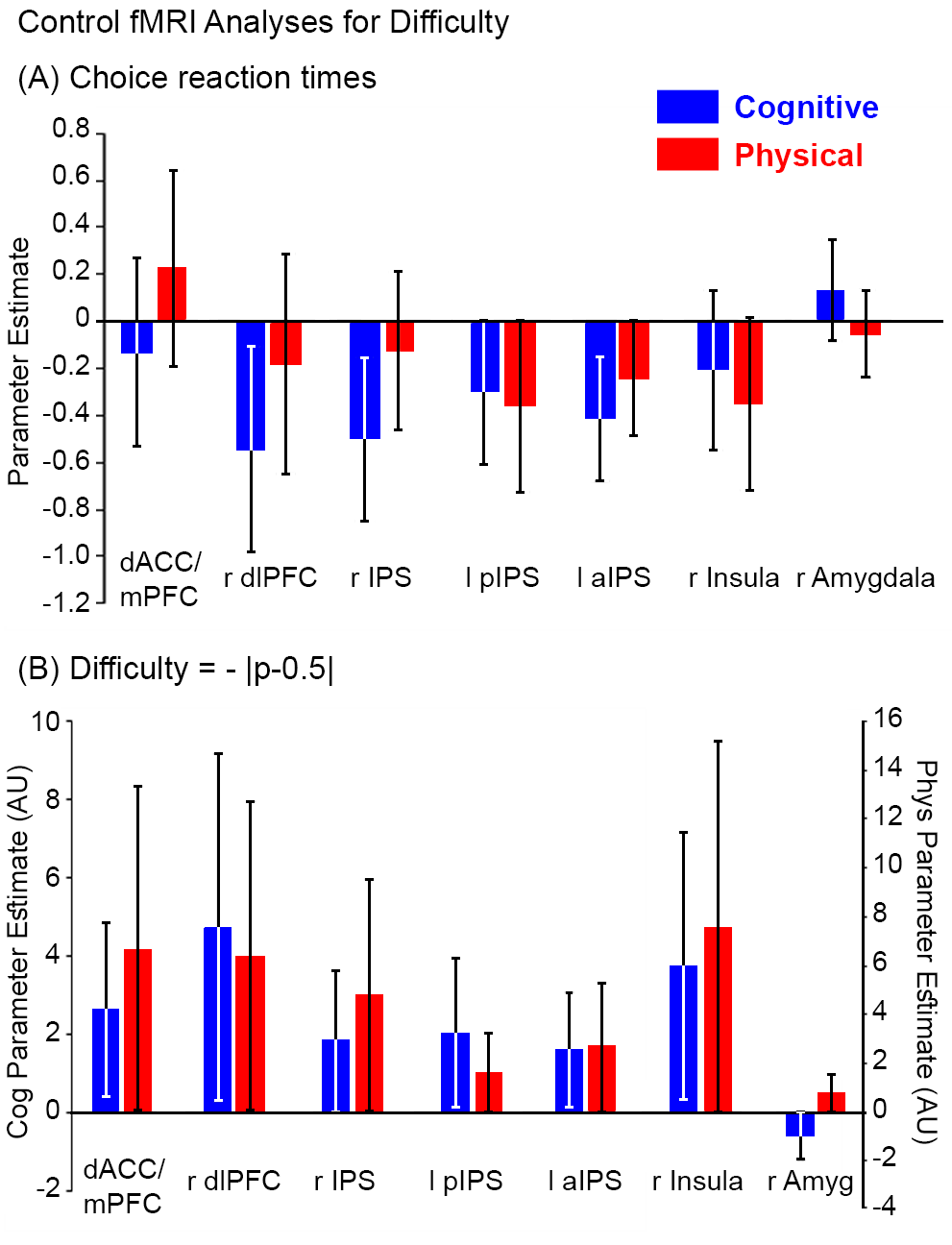

Supplement: S9 Fig — Results of control fMRI analyses for choice difficulty, using (A) choice reaction time and (B) choice probabilities from the softmax model. Each of these measures were entered into a general linear model as parametric regressors time-locked to the onset of the offer cue. None of the areas that signalled subjective value signalled choice difficulty. Underlying data for panels A–B can be found in S1 Data. (TIF) [file pbio.1002598.s009.tif]

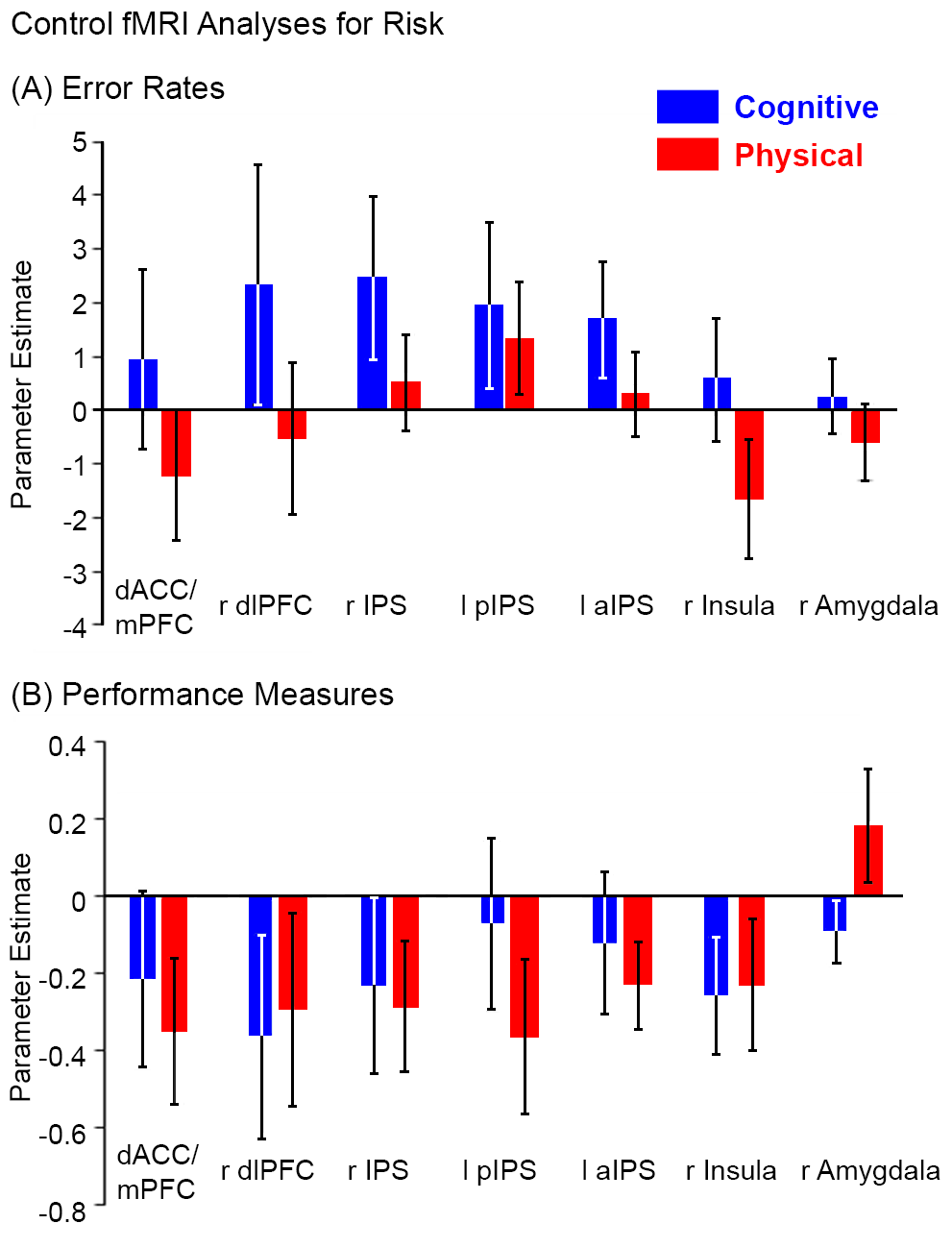

Supplement: S10 Fig — Results of control fMRI analyses for risk, using (A) error rates and (B) performance measures for each effort level in each task during the prescan training session. d’ was used as a composite measure of performance in the cognitive effort task, and the time spent within the required force window was used as an index of performance for the physical effort task. Each measure was entered into a general linear model as parametric regressors time-locked to the onset of the offer cue. None of the areas that signalled subjective value signalled risk. Underlying data for panels A–B can be found in S1 Data. (TIF) [file pbio.1002598.s010.tif]
