# Supplementary material for: Neurocomputational mechanisms underlying subjective valuation of effort costs
Source: PLoS Biol. 2017 Feb 24;15(2):e1002598. doi: 10.1371/journal.pbio.1002598 (PMC5325181; doi:10.1371/journal.pbio.1002598)
Supplement: S1 Table — Data are shown for (A) cognitive effort, (B) physical effort, and (C) the conjunction of both domains. Clusters are significant at a voxel-wise threshold of p < .05, corrected for family-wise error. Coordinates are given in MNI space. (DOCX) [file pbio.1002598.s011.docx]

**Supplementary Table S1**

*Areas sensitive to the SV difference between the chosen option and baseline, time-locked to the onset of the* ***response*** *prompt. Data are shown for* (A) *Cognitive Effort,* (B) *Physical Effort,* and (C) *the Conjunction of both domains. Clusters are significant at a voxel-wise threshold of p < .05, corrected for family-wise error. Coordinates are given in Montreal Neurological Institute (MNI) space.*

| Area | Peak voxel | *k* | *Z* value | Voxel *p* (FWE) |
| --- | --- | --- | --- | --- |
| **Cognitive Effort** | | | | |
| Cerebellum Crus II | -34 -74 -50 | 32 | 5.42 | 0.002 |
| Subgenual cingulate | 0 14 -8 | 34 | 5.30 | 0.003 |
| **Physical Effort** | | | | |
| Cerebellum Crus II | -36 -64 -44 | 212 | 5.70 | 0.000 |
| **Conjunction of Cognitive and Physical Effort** | | | | |
| Cerebellum Crus II | -36 -70 -48 | 359 | 5.06 | 0.01 |
