# Supplementary material for: Neurocomputational mechanisms underlying subjective valuation of effort costs
Source: PLoS Biol. 2017 Feb 24;15(2):e1002598. doi: 10.1371/journal.pbio.1002598 (PMC5325181; doi:10.1371/journal.pbio.1002598)
Supplement: S1 Data — (DOCX) [file pbio.1002598.s012.docx]

**Supporting Data for Figures 2, 3, 4D, 5, and S1-S6 and S8-S10.**

**Figure 2**

Mean proportion of accepted offers (SEM) as a function of Effort and Reward for the Cognitive and Physical Effort tasks.

| **Effort** | **2** | **3** | **4** | **5** | **6** |
| --- | --- | --- | --- | --- | --- |
| Cognitive | 0.96 (0.01) | 0.85 (0.03) | 0.69 (0.05) | 0.60 (0.05) | 0.54 (0.05) |
| Physical | 0.97 (0.01) | 0.90 (0.02) | 0.75 (0.04) | 0.65 (0.04) | 0.55 (0.05) |
| **Reward** | **2** | **4** | **6** | **8** | **10** |
| Cognitive | 0.40 (0.04) | 0.65 (0.05) | 0.81 (0.04) | 0.88 (0.03) | 0.91 (0.03) |
| Physical | 0.48 (0.05) | 0.67 (0.05) | 0.84 (0.04) | 0.88 (0.03) | 0.94 (0.02) |

**Figure 3 and Supplementary Figure 5**

Model comparisons using the Akaike Information Criterion (AIC) and Bayesian Information Criterion (BIC) reveal similar patterns of results. AIC and BIC values are shown for each of the 36 models compared. The winning model (with the lowest AIC or BIC values, highlighted in red) described cognitive effort discounting according to a hyperbolic function, and physical effort discounting as a parabolic function.

| **AIC** |  | **Single function** |  | **Separate functions; Single *softmax* β** | | | |  | **Separate functions; Separate *softmax* βs** | | | |
| --- | --- | --- | --- | --- | --- | --- | --- | --- | --- | --- | --- | --- |
| **Cognitive Discounting Function** | *Parabolic* | 3310.5 |  | 2664.9 | 2616.4 | 2639.2 | 2637.3 |  | 2603.7 | 2462.1 | 2425.0 | 2559.9 |
|  | *Hyperbolic* | 3165.6 |  | 2842.7 | 2553.5 | 2581.4 | 2550.8 |  | 2660.4 | 2459.0 | 2506.4 | **2367.8** |
|  | *Exponential* | 3090.8 |  | 2755.3 | 2471.5 | 2585.7 | 2579.3 |  | 2570.7 | 2447.7 | 2457.9 | 2378.3 |
|  | *Linear* | 3377.7 |  | 2836.7 | 2747.2 | 2852.8 | 2585.9 |  | 2763.6 | 2601.7 | 2662.8 | 2476.4 |
|  |  | *Identical* |  | *Linear* | *Exponential* | *Hyperbolic* | *Parabolic* |  | *Linear* | *Exponential* | *Hyperbolic* | *Parabolic* |

**Physical Discounting Function**

| **BIC** |  | **Single function** |  | **Separate functions; Single *softmax* β** | | | |  | **Separate functions; Separate *softmax* βs** | | | |
| --- | --- | --- | --- | --- | --- | --- | --- | --- | --- | --- | --- | --- |
| **Cognitive Discounting Function** | *Parabolic* | 3514.2 |  | 2970.3 | 2921.9 | 2944.7 | 2942.8 |  | 3011.0 | 2869.4 | 2832.3 | 2967.2 |
|  | *Hyperbolic* | 3369.3 |  | 3148.1 | 2859.0 | 2886.9 | 2856.3 |  | 3067.7 | 2866.3 | 2913.7 | **2775.1** |
|  | *Exponential* | 3294.5 |  | 3060.8 | 2777.0 | 2891.2 | 2884.7 |  | 2978.0 | 2855.0 | 2865.2 | 2785.6 |
|  | *Linear* | 3581.3 |  | 3142.2 | 3052.7 | 3158.3 | 2891.4 |  | 3170.9 | 3009.0 | 3070.1 | 2883.7 |
|  |  | *Identical* |  | *Linear* | *Exponential* | *Hyperbolic* | *Parabolic* |  | *Linear* | *Exponential* | *Hyperbolic* | *Parabolic* |

**Physical Discounting Function**

**Figure 4D and Supplementary Figure 8**

Mean parameter estimates (SEM) for each domain-general area as a function of: the SV difference between the chosen option and the baseline; the effort level of the offer; and the reward level of the offer. Data are shown separately for Cognitive and Physical Effort decisions.

|  | **dACC/mPFC** | **r dlPFC** | **r IPS** | **l pIPS** | **l aIPS** | **r Insula** |
| --- | --- | --- | --- | --- | --- | --- |
| **Cognitive Effort** | | | | | | |
| **SV Diff** | -0.36 (0.04) | -0.37 (0.05) | -0.31 (0.04) | -0.31 (0.03) | -0.24 (0.02) | -0.23 (0.03) |
| **Effort** | 0.13 (0.07) | 0.14 (0.08) | 0.09 (0.05) | 0.13 (0.05) | 0.10 (0.04) | 0.10 (0.05) |
| **Reward** | -0.20 (0.06) | -0.16 (0.07) | 0.16 (0.06) | -0.12 (0.05) | -0.16 (0.05) | -0.17 (0.06) |
| **Physical Effort** | | | | | | |
| **SV Diff** | -0.29 (0.04) | -0.30 (0.04) | -0.23 (0.03) | -0.18 (0.03) | -0.15 (0.03) | -0.19 (0.03) |
| **Effort** | 0.21 (0.07) | 0.19 (0.07) | 0.15 (0.05) | 0.16 (0.05) | 0.12 (0.04) | 0.13 (0.06) |
| **Reward** | -0.28 (0.08) | -0.29 (0.08) | -0.23 (0.06) | -0.14 (0.06) | -0.13 (0.05) | -0.23 (0.06) |

**Figure 5**

Mean parameter estimates (SEM) within the right amygdala for the subjective value of the cognitive and physical offer.

|  | **Amygdala** |
| --- | --- |
| Cognitive | 0.095 (0.019) |
| Physical | 0.015 (0.017) |

**Supplementary Figure 1**

Mean success rates (SEM) for the cognitive and physical effort tasks in the pre-scan training session.

| **Effort Level** | **1** | **2** | **3** | **4** | **5** | **6** |
| --- | --- | --- | --- | --- | --- | --- |
| Cognitive | 0.97 (0.01) | 0.99 (0.00) | 0.97 (0.01) | 0.96 (0.01) | 0.97 (0.01) | 0.95 (0.01) |
| Physical | 0.99 (0.01) | 1.00 (0.00) | 0.99 (0.00) | 0.99 (0.01) | 0.95 (0.02) | 0.91 (0.03) |

**Supplementary Figure 2**

Median reaction times (SEM) for decisions made in the scanner, as a function of Effort and Reward for the Cognitive and Physical Effort tasks.

| **Effort** | **2** | **3** | **4** | **5** | **6** |
| --- | --- | --- | --- | --- | --- |
| Cognitive | 0.79 (0.03) | 0.80 (0.03) | 0.79 (0.03) | 0.79 (0.03) | 0.80 (0.03) |
| Physical | 0.75 (0.02) | 0.81 (0.03) | 0.80 (0.03) | 0.83 (0.03) | 0.80 (0.03) |
| **Reward** | **2** | **4** | **6** | **8** | **10** |
| Cognitive | 0.81 (0.03) | 0.79 (0.03) | 0.82 (0.03) | 0.77 (0.02) | 0.78 (0.03) |
| Physical | 0.80 (0.03) | 0.81 (0.03) | 0.80 (0.03) | 0.81 (0.03) | 0.77 (0.03) |

**Supplementary Figure 3**

Normalised t-statistics (*β*/SE(*β*)) from the logistic regression examining the effect of Reinforcement, Effort and Reward on choice behaviour for the Cognitive and Physical Effort tasks.

|  | **Reinforcement** | **Effort** | **Reward** |
| --- | --- | --- | --- |
| Cognitive | 0.22 (0.20) | -1.92 (0.27) | 1.90 (0.27) |
| Physical | 0.10 (0.24) | -1.86 (0.27) | 1.81 (0.28) |

**Supplementary Figure 4**

Data from two participants showing mean proportion of accepted offers as a function of Cognitive and Physical Effort Level.

| **Effort Level** | **2** | **3** | **4** | **5** | **6** |
| --- | --- | --- | --- | --- | --- |
| **Participant 1** | | | | | |
| * Cognitive | 0.87 | 0.50 | 0.07 | 0.07 | 0.00 |
| * Physical | 0.93 | 0.73 | 0.70 | 0.60 | 0.53 |
| **Participant 2** | | | | | |
| * Cognitive | 1.00 | 1.00 | 0.87 | 0.73 | 0.80 |
| * Physical | 1.00 | 0.53 | 0.33 | 0.27 | 0.20 |

**Supplementary Figure 6**

Perceived demand in the Cognitive and Physical Effort tasks, as assessed with the NASA Task Load Index. Data indicate mean NASA scores (SEM) for Mental > Physical Demand. Positive values indicate greater Mental Demand.

| **Effort** | **1** | **2** | **3** | **4** | **5** | **6** |
| --- | --- | --- | --- | --- | --- | --- |
| Cognitive | 3.29 (0.60) | 3.47 (0.64) | 5.59 (0.80) | 6.88 (0.88) | 8.53 (0.98) | 10.03 (1.07) |
| Physical | -0.50 (0.48) | -1.68 (0.51) | -2.56 (0.52) | -4.09 (0.72) | -5.94 (0.82) | -7.50 (1.03) |

**Supplementary Figures 9 & 10**

Mean parameter estimates (SEM) for the domain-general and domain-specific regions, with Choice Reaction Time, Difficulty, Error Rates, and Performance entered as regressors for Cognitive and Physical Effort choices.

|  | **dACC/mPFC** | **r dlPFC** | **r IPS** | **l pIPS** | **l aIPS** | **r Insula** | **r Amygdala** |
| --- | --- | --- | --- | --- | --- | --- | --- |
| **Choice Reaction Time** | | | | | | | |
| Cog | -0.13 (0.40) | -0.55 (0.44) | -0.50 (0.35) | -0.30 (0.31) | -0.41 (0.26) | -0.21 (0.34) | 0.13 (0.22) |
| Phys | 0.23 (0.41) | -0.18 (0.47) | -0.13 (0.34) | -0.36 (0.37) | -0.24 (0.25) | -0.35 (0.37) | -0.05 (0.19) |
| **Difficulty** | | | | | | | |
| Cog | 2.63 (2.20) | 4.74 (4.43) | 1.85 (1.80) | 2.02 (1.94) | 1.60 (1.49) | 3.75 (3.43) | -0.84 (0.86) |
| Phys | 6.72 (6.66) | 6.42 (6.36) | 4.82 (4.77) | 1.64 (1.60) | 2.70 (2.67) | 7.64 (7.60) | 0.82 (0.81) |
| **Error Rates** | | | | | | | |
| Cog | 0.95 (1.68) | 2.33 (2.23) | 2.46 (1.52) | 1.94 (1.54) | 1.68 (1.10) | 0.58 (1.15) | 0.25 (0.70) |
| Phys | -1.22 (1.22) | -0.54 (1.41) | 0.52 (0.89) | 1.33 (1.05) | 0.29 (0.78) | -1.65 (1.11) | -0.60 (0.72) |
| **Performance** | | | | | | | |
| Cog | -0.22 (0.23) | -0.36 (0.26) | -0.23 (0.23) | -0.08 (0.22) | -0.12 (0.18) | -0.26 (0.15) | -0.09 (0.08) |
| Phys | -0.35 (0.19) | -0.29 (0.25) | -0.29 (0.17) | -0.37 (0.19) | -0.23 (0.12) | -0.23 (0.17) | 0.18 (0.15) |
